# Overview of the role of robots in upper limb disabilities rehabilitation: a scoping review

**DOI:** 10.1186/s13690-023-01100-8

**Published:** 2023-05-08

**Authors:** Khadijeh Moulaei, Kambiz Bahaadinbeigy, Ali Akbar Haghdoostd, Mansour Shahabi Nezhad, Abbas Sheikhtaheri

**Affiliations:** 1 Medical Informatics Research Center, Institute for Futures Studies in Health, Kerman University of Medical Sciences, Kerman, Iran; 2 HIV/STI Surveillance Research Center, WHO Collaborating Center for HIV Surveillance, Institute for Futures Studies in Health, Kerman University of Medical Sciences, Kerman, Iran; 3 Department of Physical Therapy, Faculty of Allied Medicine, Kerman University of Medical Sciences, Kerman, Iran; 4 Department of Health Information Management, School of Health Management and Information Sciences, Iran University of Medical Sciences, Tehran, Iran

**Keywords:** Robots, Rehabilitation, Upper limb, Disabilities, Digital Health

## Abstract

**Background:**

Neuromotor rehabilitation and improvement of upper limb functions are necessary to improve the life quality of patients who have experienced injuries or have pathological outcomes. Modern approaches, such as robotic-assisted rehabilitation can help to improve rehabilitation processes and thus improve upper limb functions. Therefore, the aim of this study was to investigate the role of robots in upper limb disability improvement and rehabilitation.

**Methods:**

This scoping review was conducted by search in PubMed, Web of Science, Scopus, and IEEE (January 2012– February 2022). Articles related to upper limb rehabilitation robots were selected. The methodological quality of all the included studies will be appraised using the Mixed Methods Appraisal Tool (MMAT). We used an 18-field data extraction form to extract data from articles and extracted the information such as study year, country, type of study, purpose, illness or accident leading to disability, level of disability, assistive technologies, number of participants in the study, sex, age, rehabilitated part of the upper limb using a robot, duration and frequency of treatment, methods of performing rehabilitation exercises, type of evaluation, number of participants in the evaluation process, duration of intervention, study outcomes, and study conclusions. The selection of articles and data extraction was made by three authors based on inclusion and exclusion criteria. Disagreements were resolved through consultation with the fifth author. Inclusion criteria were articles involving upper limb rehabilitation robots, articles about upper limb disability caused by any illness or injury, and articles published in English. Also, articles involving other than upper limb rehabilitation robots, robots related to rehabilitation of diseases other than upper limb, systematic reviews, reviews, and meta-analyses, books, book chapters, letters to the editor, and conference papers were also excluded. Descriptive statistics methods (frequency and percentage) were used to analyses the data.

**Results:**

We finally included 55 relevant articles. Most of the studies were done in Italy (33.82%). Most robots were used to rehabilitate stroke patients (80%). About 60.52% of the studies used games and virtual reality rehabilitate the upper limb disabilities using robots. Among the 14 types of applied evaluation methods, “evaluation and measurement of upper limb function and dexterity” was the most applied evaluation method. “Improvement in musculoskeletal functions”, “no adverse effect on patients”, and “Safe and reliable treatment” were the most cited outcomes, respectively.

**Conclusions:**

Our findings show that robots can improve musculoskeletal functions (musculoskeletal strength, sensation, perception, vibration, muscle coordination, less spasticity, flexibility, and range of motion) and empower people by providing a variety of rehabilitation capabilities.

**Supplementary Information:**

The online version contains supplementary material available at 10.1186/s13690-023-01100-8.

## Introduction

Impaired upper limb (UL) functions to restrict the performance of activities of daily living, limit social participation [1], specifically decrease the independence of affected individuals and reduce patients’ quality of life [2, 3]. In upper limb disabilities, patients develop musculoskeletal problems such as paresis, pain, loss of sensation, and spasticity in different parts of the upper limb and so these problems can have manifold consequences in the daily lives of those impacted. These include a decreased capacity to carry out primary self-care tasks and to accomplish life-roles, which can affect emotional, mental, and psychological wellbeing [4]. Patients with upper limb disabilities need rehabilitation to improve their musculoskeletal status [5].

Neuromotor rehabilitation and recovery of upper extremity functions are necessary to improve the life quality of individuals who have suffered injuries, disabilities or have pathological outcomes, where it is favorable to raise the development of activities of daily living [6]. However, the conventional rehabilitations done by one manual-assisted movement training created by physiotherapists suffers from a lot of inherent restrictions, such as heavy labor severity, long-time consumption, lack of repeatability, low patients participation, and their low motivation to perform rehabilitation exercises [7]. Also, in conventional therapy, the accessibility of therapists, the duration of therapy sessions, and the high cost of rehabilitation tools are all considered parameters that impact on both therapists and patients [8].

New therapeutic methods have been presented to rehabilitate and improve upper limb function, and such methods are robotic rehabilitation [1]. Severe repetitions of harmonized motor activities by robots establish an important burden for the therapists who help patients. Moreover and due to economic reasons, the duration of preliminary rehabilitation is getting shorter and shorter [9]. However, some studies have pointed out that repetitive and high-severity exercises can specifically contribute to the functional recovery of the affected upper limb movement [10]. Rehabilitation robots are able of decreasing the burden on therapists by replace human intervention and preparing desire therapies that accomplish the following primary principles of upper limb rehabilitation: iteration, high severity, and task particularly [11]. Moreover, the functional impetus of a patient can be activated to raise strenuous participation and improve cognitive functions. The physical factors and treatment data can be stored and analyzed by the sensing system, and that can provide a realistic basis for optimization training technique and speed up the recovery process [7]. In addition, major boost has been given by the extension of such devices to clinical care medical and rehabilitation centers [1, 2].

Therefore, multiple benefits can be considered for rehabilitation robots : they can produce high-quality repetitive movements and increase rehabilitation strength and intensity; they can offer many types of exercises for therapists to choose from; they supply a man-machine interaction that allows for objective measurement of advancement, which itself can status modifications in interaction by changing control parameters [12]. On the other hand, task-oriented training is one of the other capabilities of rehabilitation robots, which is known as the dominant and most effective method for motor rehabilitation of upper limb function [13]. Task-based approaches in which the patient is assisted to perform a specific prescribed movement, such as lifting an object with the hand, show promising results compared to conventional exercises based on passive movement of the impaired joints in the restriction of their range of motion [14].

Robots can also be used in homes, clinics and medical centers. Patients can do the rehabilitation exercises prescribed by the therapist with the help of the robot at home without having to visit the treatment center frequently. Successful robot-assisted rehabilitation at home can facilitate intensive therapy, facilitate in-person or virtual therapy visits, preferably at low cost, and motivate patients to participate in supervised or unsupervised therapeutic activities at necessary levels for motor learning and generalization to occur [15]. Moreover, in clinics, rehabilitation robots can decrease the burden of therapists by automating tedious and labor-intensive treatment and by adapting to the particularized needs of targeted individuals [16]. For example, the hand robot skeleton designed by Wege et al., [17] can move patients’ fingers skillfully and repeatedly compared to a tired therapist training patients with manual labor.

To our knowledge, no systematic or scoping review has been performed on robots to rehabilitate upper limb disabilities. Only a few systematic reviews have been done in the areas of classification of interactive wearable systems for monitoring body movement and posture during upper body rehabilitation, estimating the wearability of the wearable devices [18], presenting state of the art in sensor fusion applied in applications for hand rehabilitation [19], evaluating the role of serious games in upper limb rehabilitation, identifying common procedures and exercises as well as technology patterns [20], evaluating the effectiveness of upper limb wearable technology to improve activity and engagement in stroke survivors [21] and determining the effects of robot-assisted treatment on motor-functional improvement in stroke patients [22]. So, the aim of the present study was to investigate the role of robots in upper limb disability improvement and rehabilitation. In this study, we identify the most common diseases or complications leading to upper limb disability for which robots have been used for rehabilitation. Moreover, we identify the most important technologies that can be used along with rehabilitation robots, the types of evaluation methods of rehabilitation robots and the outcomes of robot use for individuals with upper limb disabilities.

## Materials and methods

In the current study, we used the PRISMA scoping reviews checklist for selecting studies and reporting the results [23].

### Search strategy and information sources

To find articles related to the rehabilitation of upper limb disabilities using robots, four databases, PubMed, Web of Science, Scopus, and IEEE were searched. In order to search these databases, the keywords related to the upper limb, rehabilitation, and robots were used. Relevant Medical Subject Heading (MeSH) Keywords, spelling differences and synonyms were included and altered as suitable for each database. Then, the below search strategy was developed by KB, ASH, and KHM and finally approved by AH.

((upper extremity disability OR upper limb disability) AND (rehabilitation OR telerehabilitation) AND (robot OR robotics))

**Fig. 1:**
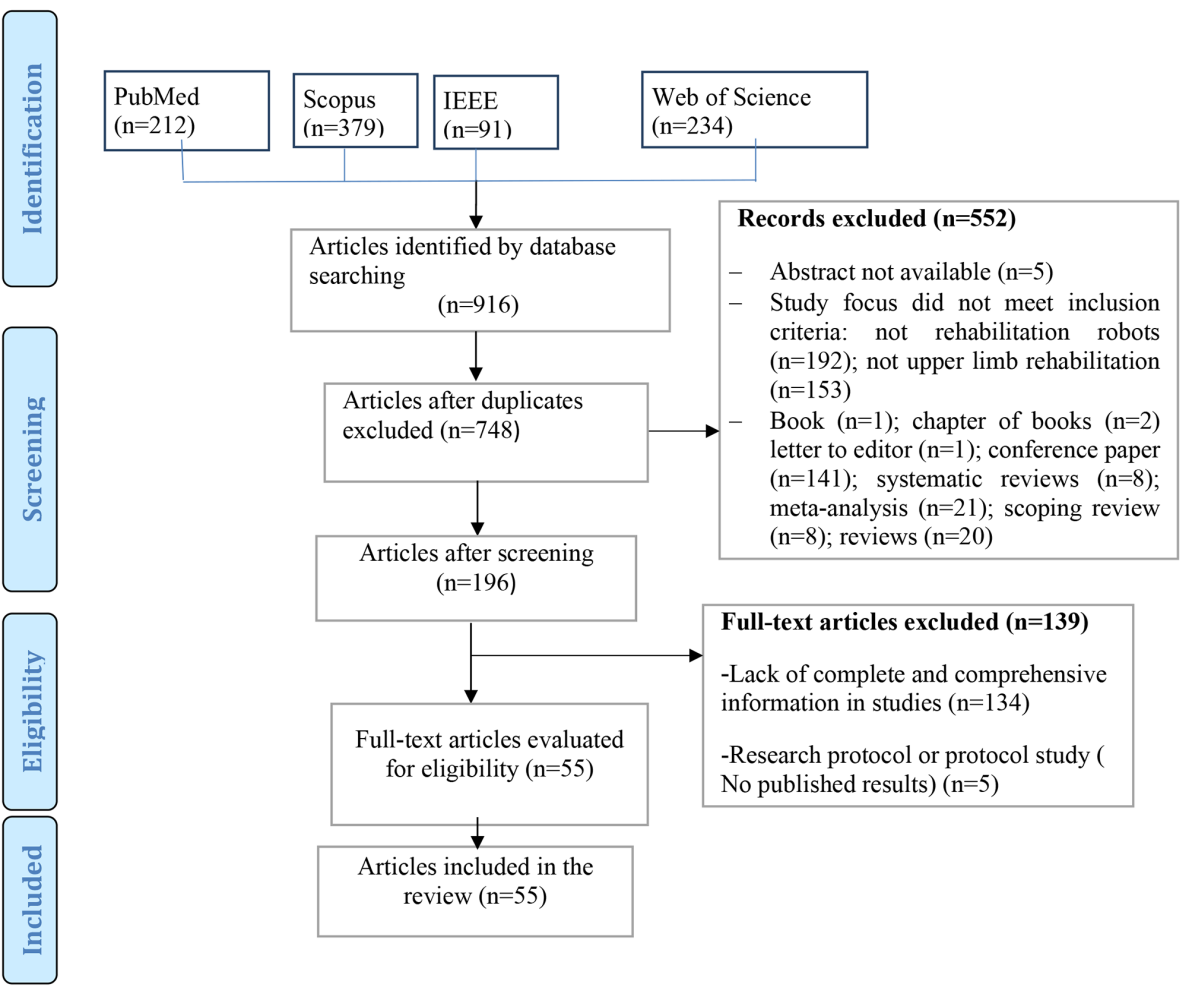
Study selection process

Articles addressing upper limb rehabilitation robots were included in to study. The PRISMA diagram is demonstrated in Fig. 1.

### Eligibility criteria

Inclusion and exclusion criteria are presented below.

#### Inclusion criteria

1. Articles involving upper limb rehabilitation robots.
2. Articles about upper limb disability caused by any illness or injury.
3. Studies involving rehabilitation robots designed for all ages.
4. Robotic devices used with any assistive technologies (such as telerehabilitation systems and games based on virtual reality, etc.)
5. Articles published in English.
6. Time of publication between January 2012– February 2022.

It should be noted due to the rapid development of technologies and rehabilitation robots, we limited our investigation to the last ten years. We wanted to identify the latest and newest technologies that can be used along with rehabilitation robots, evaluation methods and the outcomes of these robots in recent years. Xue et al. [24], believed that rehabilitation robotics is a relatively young field growing rapidly with increasing penetration into therapeutic environments. Therefore, the newer dimensions of this rehabilitation tool should always be considered. In addition, the number of articles published in the last ten years was sufficient to answer the questions of this research. Therefore, this scoping review included articles published from January 2012– February 2022.

#### Exclusion criteria

1. Articles involving other than upper limb rehabilitation robots.
2. Robots related to rehabilitation of diseases other than upper limb.
3. Systematic reviews, reviews, and meta-analysis.
4. Books.
5. Book chapters.
6. Letter to the editor.
7. Conference abstracts.
8. Research protocol or protocol study.

### Study selection

First, abstracts of all related articles were retrieved from four databases: PubMed, Web of Science, Scopus, and IEEE, and imported into EndNote software by KHM. Duplicate articles were removed. After studying the title, and abstract, the relevant articles were selected according to the inclusion and exclusion criteria by KHM and ASH. Then, all studies included in the study were reviewed and finalized by KB and AH. In disagreement, the final decision on each article is decided by discussion among the study team members. Finally, the full text of the articles was studied to extract data and information from KHM and ASH.

### Critical appraisal of individual sources of evidence

Critical evaluation of data was done by ASH and KHM independently using Mixed Methods Appraisal Tool (MMAT, version 2018: Hong et al., 2018). Disagreement between authors (n = 4 articles) was resolved by discussion between two other authors (KB and AH) until reaching an agreement. We evaluated studies according to the MMAT criteria according to the selected category. The latest version of the MMAT offers a descriptive quality appraisal instead of a summative numerical score. Answer options in all study categories include “yes”, “no” and “can’t tell”. The answer “can’t tell” indicates that not enough information was reported in the study for a “yes” or “no” answer. A “can’t tell” rating may indicate a need to search for companion studies or contact study authors for more information [25].

### Data charting process and data items

Data were extracted using a predetermined form. The validity of this form was confirmed by two specialists in medical informatics and software engineer, a physiotherapist, and a specialist in health information management. This data extraction form also includes fields such as year, country, type of study, purpose, illness or accident leading to disability (Table 1), level of disability (Table 2), assistive technologies (telerehabilitation, virtual reality or games) (Table 1 and more details in Appendix B), number of participants in the study, sex, age (Table 3 and more details in B)), rehabilitated part of the upper limb using a robot (Table 4), duration and frequency of treatment, methods of performing rehabilitation exercises, type of evaluation, number of participants in the evaluation process (Table 5 and more details in Appendix B), study outcomes (Table 6 and more details in Appendix B) and study conclusions (more details in Appendix B).

**Table 1:** Summary of results from reviewed articles

Ref	Country	Year	Study type	Study aim	Illness or accident leading to disability	Level of disability	Use of telerehabilitation	Use games and virtual reality
Hwang [66]	Republic of Korea	2012	Randomized controlled trials (RCT)	Evaluating robot-assisted hand and finger rehabilitation in stroke patients	Stroke	Fingers		√
Carpinella [26]	Italy	2012	Pilot study	Comparing two reaching tasks (RT) and objects’ reaching and manipulation (RMT) protocols for upper extremity robot-based rehabilitation in MS patients	Multiple sclerosis (MS)	Upper limb		
Hu [56]	USA	2013	Experimental study	Investigating the effectiveness of robot-assisted upper limb training in stroke patients	Stroke	Fingers and wrist		√
Squeri [57]	Italy	2014	Pilot study	Developing a novel therapeutic protocol aimed at restoring wrist functionality in chronic stroke patients	Stroke	Wrist and forearm		√
Sale [76]	Italy	2014	RCT	Evaluating the effects of robot-assisted hand therapy compared to intensive occupational therapy in stroke patients	Stroke	Hand		
Sale [27]	Italy	2014	Before and after	Determining the short-term and long-term changes in the motor performance of patients with chronic hemiparesis after using a rehabilitation robot	Chronic hemiparesis	Upper limb		
Klamroth-Marganska [70]	Switzerland	2014	RCT	Evaluation of the effect of a skeleton robot in training the injured arm compared to conventional treatment	Stroke	Arm, elbow and shoulder		√
Hsieh [58]	Taiwan	2014	Observational cohort study	Investigating the predictors of minimal clinically important changes on outcome measures after robot-assisted therapy	Stroke	Wrist and forearm		
Pennati [28]	Italy	2015	RCT	Investigating the effect of combining a short robotic exercise and chemical neurolysis in reducing spasm and improving function in patients with stroke	Stroke	Upper limb		
McCabe [29]	USA	2015	RCT	Investigating the effect of using robotics on motor learning of the upper limbs of chronic and injured stroke survivors	Stroke	Upper limb		
Chen [59]	China	2015	Not mentioned	Design and development of a cable wrist robotic rehabilitation device for motor training or assisting people with motor disabilities in the upper limb.	Stroke	Wrist and arm		√
Vanmulken [71]	Netherlands	2015	Feasibility study	Investigating the feasibility of tactile robot technology in improving arm and hand performance and skill in people with cervical spinal cord injury	Spinal cord injury (C-SCI)	Arm and hand		
Gilliaux [30]	Belgium	2015	RCT	Evaluating the effectiveness of robot-assisted therapy in children with cerebral palsy	Cerebral Palsy	Upper limb		√
Taveggia [31]	Italy	2016	RCT	Evaluating the effectiveness of robotic-assisted movement and activity in upper limb rehabilitation in hospitalized patients after stroke	Stroke	Upper limb		√
Biggar [77]	United Kingdom	2016	Feasibility Study	Design and development of a wearable robotic glove to assist in the rehabilitation of patients at home	Stroke	Hand		
Orihuela-Espina [78]	Mexico	2016	RCT	Determining the effectiveness of robot-based treatments in the motor improvement of stroke patients	Stroke	Hand		
Song [32]	China	2016	Not mentioned	Development and design of a robot for upper limb telerehabilitation after a stroke	Stroke	Upper limb	√	√
Vanoglio [79]	Italy	2017	RCT	Evaluating the feasibility and effectiveness of hand and arm rehabilitation with the help of a robot in subacute hemiplegic patients	Stroke	Hand		
Trujillo [33]	Italy	2017	Not mentioned	Assessing the relationship between quantitative electroencephalography (QEEG) measures and motor outcome in chronic stroke patients undergoing a robot-assisted rehabilitation program to predict motor recovery	Stroke	Upper limb		
Saita [34]	Japan	2017	Pilot study	Investigating the effects of robot-assisted rehabilitation and botulinum toxin in the treatment of paretic arm with spasticity in stroke patients	Stroke	Upper limb		
Nam [80]	Hong Kong	2017	RCT	Investigating the effects of robotic-assisted rehabilitation and training on the upper limbs of people with chronic stroke	Stroke	Hand and elbow		
McKenzie [60]	USA	2017	Cross sectional	Validation and evaluation of the effect of a rehabilitation robot in improving arm motor function after stroke	Stroke	Wrist and fingers		√
Kim [72]	USA	2017	RCT	Comparison of long-term effects of external and internal focus after robot-assisted arm training	Stroke	Arm and shoulder		√
Bishop [67]	Columbia	2017	Pilot Study	Investigating the effect of training with a robotic system on paralysis and hand function in hemiparesis patients	Hemiparesis	Fingers		√
Housley [61]	USA	2017	Pilot study	Investigating the improvement of upper limb function and quality of life due to the use of a robot skeleton	Stroke	Wrist	√	√
Hsieh [35]	Taiwan	2017	RCT	Investigating the therapeutic effects of robotic priming on daily function, movement disorders, and quality of life in stroke patients	Stroke	Upper limb		
Gandolfi [36]	Italy	2018	RCT	Comparison of the effect of robot-assisted hand training on muscle activity, hand skills, and upper limb dysfunction	MS	Upper limb		√
Lee [74]	Korea	2018	Pilot study	Design, development and evaluation of a shoulder joint tracking module for upper limb rehabilitation robots	Stroke	Shoulders		
Germanotta [37]	Italy	2018	Cross-sectional	Evaluation of validity, capability and reliability of a robotic device for upper limb rehabilitation	Stroke	Upper limb		
Kim [38]	Korea	2018	Pilot stud	Evaluation of the effects of therapeutic exercise with a robot in improving the upper limb in patients with chronic stroke	Stroke	Upper limb		√
Villafañe [68]	Italy	2018	RCT	Evaluation of the effect of robot and occupational therapy in motor improvement of stroke patients	Stroke	Fingers, shoulder, and arm		√
Palermo [62]	Italy	2018	Before and after	Evaluation of the effects of robotic rehabilitation on ten subacute stroke survivors	Stroke	Shoulders, elbow, wrist		√
Iwamoto [39]	Japan	2018	Not mentioned	Determining the effects of using single-joint hybrid auxiliary limb in upper limb rehabilitation of stroke patients	Stroke	Upper limb		
Kim [75]	South Korea	2019	RCT	Investigating the therapeutic effects of a shoulder robot on hemiplegic shoulder pain after stroke	Stroke	Shoulder		√
Dehem [40]	Belgium	2019	RCT	Evaluating the effectiveness of upper limb robotic-assisted treatment as an alternative to conventional treatment in the rehabilitation of stroke patients	Stroke	Upper limb		√
Hung [41]	Taiwan	2019	RCT	Investigating the effects of combined unilateral and bilateral hybrid therapy compared to robot-assisted therapy in patients with chronic stroke	Stroke	Upper Limb		
Conroy [42]	USA	2019	RCT	Investigating the effectiveness of robot therapy on motor outcomes of patients with moderate to severe arm disability with chronic stroke	Stroke	Upper limb		√
Bonanno [43]	Italy	2019	Case series study	Investigating motor-functional improvement in multiple sclerosis patients after robot-assisted rehabilitation	MS	Upper limb		√
Leem [44]	Republic of Korea	2019	Retrospective study	Determining the effect of robot therapy on stroke patients according to the demographic and clinical characteristics of these patients	Stroke	Upper limb		√
Kim [45]	South Korea	2019	Before and after	Investigating the effect of sensory stimulation and upper limb function of stroke patients after rehabilitation with the help of robots and virtual reality	Stroke	Upper limb		√
Tartamella [46]	Italy	2020	Case study	Evaluation of the usefulness of a robotic intensive neural rehabilitation program to improve functional independence in a 57-year-old patient with BRN	Brainstem radionecrosis (BRN)	Upper limb		√
Solaro [47]	Italy	2020	RCT	Comparing robot-assisted training based on tactile or sensorimotor training in the rehabilitation of upper limb disabilities in multiple sclerosis patients	MS	Upper limb		√
Picelli [63]	Italy	2020	RCT	Evaluation of the effects of robot-assisted arm therapy in patients with distal radius injury	Distal radius fracture	Wrist and forearm		
Kuo [69]	Taiwan	2020	Case series study	Investigating the effects of robot therapy in improving the upper limb disabilities of patients with cerebral palsy	Cerebral Palsy (CP)	Fingers	-	√
Aprile [48]	Italy	2020	RCT	Investigation of shoulder pain, motor function, and quality of life in stroke patients after upper limb rehabilitation after robotic or conventional treatment	Stroke	Upper limb		√
Aprile [49]	Italy	2020	Before and after	Evaluation of the effect of using three robots and a sensor-based system in the rehabilitation of upper limb disabilities	Stroke	Upper limb		√
Bouteraa [64]	Egypt	2020	Case study	Designing and developing a new robotic system for the rehabilitation of the upper extremities	Stroke	Arm, wrist, forearm		√
Kim [50]	US	2020	RCT	Investigating the effects of following instructions on upper limb movement status in chronic stroke survivors after using a rehabilitation robot	Stroke	Upper limb		√
Bui [51]	USA	2021	Cross-sectional	Investigating the effects of robotic rehabilitation in improving cognitive and movement disorders in adults with HIV and stroke	Stroke	Upper limb		√
Flynn [52]	Australia	2021	Not mentioned	Investigating the stability of treatment and rehabilitation of the upper limb with the help of a robot in stroke survivors	Stroke	Upper limb		
Terranova [53]	Brazil	2021	RCT	Investigating the difference between robot-assisted therapy and restriction-induced movement therapy after using a rehabilitation program by chronic stroke patients.	Stroke	Upper limb		
Shi [65]	China.	2021	Before and after	Investigating the clinical effectiveness of a soft robotic hand in fingers, wrist and elbow rehabilitation	Stroke	Fingers, wrist and elbow		
Chen [73]	China	2021	RCT	Investigating the effects of robot-assisted arm training on arm motor performance, one-sided spatial neglect, social participation and daily life activities after stroke	Stroke	Arm		√
Qu [54]	China	2021	RCT	Investigating the effect of using robot-assisted training on upper limb function in stroke patients	Stroke	Upper limb		√
Abd [55]	Saudi Arabia	2022	RCT	Investigating the effects of rehabilitation exercises provided through games and robots on motor functions and upper limb spasticity in individuals with chronic stroke.	Stroke	Upper limb		√

**Table 2:** Different levels of upper limb disability

Different levels of upper limb disability	
Level of disability (references)	References frequency
Entire upper limb [26–55]	30
Wrist [56–65]	10
Fingers [56, 60, 65–69]	7
Arm [59, 64, 68, 70–73]	7
Shoulder [62, 68, 70, 72, 74, 75]	6
Hand [71, 76–80]	6
Elbow [62, 65, 70, 80]	4
Forearm [57, 58, 63, 64]	4

**Table 3:** Information of patients participating in evaluation processes

Variables		References	References frequency
**Sex**	**Male**	[46, 77]	2
	**Female**	[43]	1
	**Male and female**	[26–29, 31–42, 44, 45, 47–58, 60–63, 65–76, 78–80]	49
**Participant age**	**<=18**	[30, 67, 69, 76]	4
	**> 18**	[26–29, 31, 33–51, 53–60, 62, 63, 65–68, 70–73, 75, 76, 78–80]	47
**Duration of treatment**	**< 1 month**	[26, 28, 32, 33, 35, 39, 47, 49, 51, 54, 56, 58, 60, 62–64, 66, 68, 72, 73, 75]	21
	**1–2 month**	[29–31, 34, 36, 40, 43–46, 50, 53, 55, 57, 61, 65, 67, 69, 71, 76, 78–80]	23
	**>= 3 month**	[27, 42, 48, 52, 70]	5

**Table 4:** Rehabilitated part of the upper limb using a robot

Upper limb pasts(references)	References frequency
Entire upper limb [27–33, 37, 40, 45, 49, 51, 52, 54, 55]	15
Wrist [35, 44, 46, 53, 56–65]	14
Fingers [36, 43, 44, 46, 56, 60, 65–69, 79]	12
Shoulder [42, 46, 48, 53, 62, 68, 70, 72, 74, 75, 81]	11
Arm [26, 50, 59, 64, 68, 70–73]	9
Elbow [34, 39, 42, 46, 53, 62, 65, 70, 80]	9
Hand [47, 67, 71, 77, 78, 80]	6
Forearm [41, 57, 58, 63, 64]	5
Hand [76, 79]	2

**Table 5:** Types of evaluation in upper limb rehabilitation robots

Evaluation types	Evaluation Methods/tools(references)		References frequency	All References for evaluation types	The total frequency of types of evaluation based on the number of references
**Evaluation and measurement of upper limb function and dexterity**	Fugl-Meyer Upper Extremity score (FMA-UE) [27–29, 33–37, 41, 48–50, 53–55, 57, 58, 60, 61, 65–67, 69, 70, 72, 73, 75, 76, 78, 80]		30	[26–31, 33–51, 53–76, 78–80]	52
	Barthel Index (BI) [34, 37, 39, 48, 49, 60, 68, 73, 75, 79]		10		
	Modified Ashworth Scale (MAS) [28, 31, 34, 39, 44, 46, 55, 56, 65, 68, 80]		11		
	Box and Block Test (BBT) [27, 28, 30, 35, 38, 40, 51, 58, 60, 65, 69]		11		
	Wolf Motor Function (WMFT) [40, 42, 50, 53, 56, 57, 61, 70, 72, 80]		10		
	Action Research Arm Test (ARAT) [26, 34, 37, 47, 55, 56, 60, 65, 80]		9		
	Functional Independence Measure (FIM) [28, 31, 34, 35, 39, 44, 46, 80]		8		
	Active or Passive Range Of Motion (ROM) [55, 57, 61, 63, 64, 75]		6		
	Nine-Hole Peg Test (9HPT) [26, 36, 38, 43, 47, 79]		6		
	Stroke Impact Scale (SIS) [35, 40, 60, 61]		5		
	Motor Activity Log (MAL) [34, 39, 58]		3		
	Dynamic Surface Electromyography (DSEMG) [28, 64, 78]		3		
	ABILHAND-Kids [30, 69]		2		
	National Institutes of Health Stroke Scale (NIHSS) [68, 75]		2		
	Quality of Upper Extremity Skills Test (QUEST) [30, 67]		2		
	Jebsen-Taylor Hand Function Test (JTHFT) [45, 67]		2		
	Physical health Composite Score (PCS) [48]		1		
	Modified Rankin Scale (MRS) [35]		1		
	Arm Motor Ability Test (AMAT) [29]		1		
	Semi-structured interview to assess arm function [36]		1		
	Patient-Rated Wrist and Hand Evaluation (PRWHE) [63]		1		
	Arm-Hand Function (AHF) [71]		1		
	Pediatric Evaluation of Disability Inventory (PEDI) [67]		1		
	Performance Oriented Mobility Assessment (POMA) [46]		1		
	Reliable Change Index (RCI) [46]		1		
	Symbol Digit Modalities Test (SDMT) [49]		1		
	Total active mobility (TAM) [34]		1		
	Disability Assessment Scale (DAS) [34]		1		
	Pegboard test [66]		1		
	Intrinsic Motivation Inventory (IMI) [71]		1		
	Spinal Cord Independence(SCI) [71]		1		
	Assisting Hand Assessment (AHA) [67]		1		
	World Health Organization Disability Assessment Schedule (WHODAS) [73]		1		
	Manual Function Test (MFT) [44]		1		
	Disabilities of Arm, Shoulder, and Hand (DASH) [68]		1		
	Shoulder Disability Questionnaire (K-SDQ) [75]		1		
	Sensors [59, 74]		1		
	Reflective markers [62]				
	Grooved Pegboard Test (GP) [51]		1		
**Range and motor skills and functional strength of the hand**	Motricity Index (MI) [31, 36, 39, 48, 49, 76, 79]		7	[31, 36, 38, 39, 48, 49, 51, 55, 63, 69, 76, 79]	12
	Grip Strength Test [38, 39, 51, 55, 63, 69, 79]		7		
	Medical Research Council Scale for Muscle Strength (hand flexor and extensor muscles) (MRC) [46, 76]		2		
	Pinch Strength [79]		1		
	Quality of Movement (QOM) [39]		1		
	Amadeo® hand muscle strength: Measures of muscle strength using the robotic device [36]		1		
**Neuropsychological assessment**	Mini-Mental State Examination (MMSE) [34, 39, 44, 46, 54]		5	[34, 39, 44–46, 48, 49, 54, 71, 73]	11
	Stroop Color and Word Test (SCWT) [45, 49]		2		
	Mental health Composite Score (MCS) [48]		1		
	Behavioral Inattention Test (BIT) [73]		1		
	Credibility/Expectancy Questionnaire (CEQ) [71]		1		
	Catherine Bergego Scale (CBS) [73]		1		
	Rey Osterrieth complex figure test (ROCF) [49]		1		
	Symbol Digit Modalities Test (SDMT) [49]		1		
	Oxford Cognitive Screen (OCS) [49]		1		
	Digit Span Task [49]		1		
	Tower of London test [49]		1		
	Trail making test (TMT) [45]		1		
	Self-Depression Scale (SDS) [34]		1		
**Quality of life**	Stroke Impact Scale (SIS) [35, 40–42, 60, 61]		6	[28, 35, 36, 40–42, 48, 60, 61]	9
	Short Form Health Survey (SF-36) [48]		1		
	Nottingham Extended Activities of Daily Living (NEADL) [41]		1		
	Multiple Sclerosis Quality of Life-54 (MSQOL-54) [36]		1		
	Quality of Life (Euro-QOl) [28]		1		
**Lab-based clinical and kinematic**	Magnetic Resonance Imaging (MRI) [46, 60]		2	[34, 43, 46, 60]	4
	Functional magnetic resonance imaging (fMRI) [43]		1		
	Functional near infrared spectroscopy (fNIRS) [34]		1		
**Severity of pain**	Douleur Neuropathique 4 (DN4) [48]		1	[31, 48, 68]	3
	Numeric Rating Pain Scale (NRPS) [31, 48]		2		
	visual analog scale (VAS) [68]		1		
**Reliability**	Force and position sensors [32]		1	[32, 37]	2
	Based on the time required to complete the task, the average velocity of the device during the test, the Global length of the path travelled by the subject during center-out movements, the line integral of the force along the path described by the patient, and the amount of total work directed towards the target [37]		1		
**Efficiency**	Force and position sensors [32]		1	[32, 79]	2
	Motricity Index (MI) [79]		1		
	Nine Hole Peg Test [79]		1		
	Grip and Pinch test [79]		1		
	The quick version of the Disabilities of the Arm, Shoulder, and Hand (Quick-DASH) [79]		1		
**Feasibility of the use of the system**	Motion analysis of the fingers both with and without the device [77]		1	[77, 79]	2
	- Assessment of the side effects by reporting any adverse events occurring during the study by the physiotherapist in regard to the use of Gloreha Professional [79] -Assessment of the level of operator difficulty for the physiotherapist in managing the device by visual analogue scale (VAS) [79]		1		
**Cost analysis**	- Costs calculation in terms of the time required by healthcare personnel, using the average cost per hour of a physiotherapist per total number of rehabilitation treatments per patient and in terms of the time required by a physiotherapist to take care that the robotic device working correctly during the sessions [79]		1	[79]	1
**Tremor Severity Scale**	A clinical rating scale [36]		1	[26, 36]	2
	Tremor Severity Scale (TSS) [26]		1		
**Adherence to rehabilitation exercises**	number of treatment robot sessions and the duration of treatment sessions using the robot over time [52]		1	[52]	1
**Usability Testing**	Usefulness, Satisfaction and Ease-of-use(USE) Questioner [71]		1	[71]	1
**Patient Satisfaction**	Questionnaire	Usefulness, Satisfaction and Ease-of-use questionnaire (USE) [71]	1	[71]	1

### Data collation process

The information extracted from the articles was re-examined by KB and finally approved by AH. In case of disagreement, the consensus was achieved by the review of the members of the study team. It should be noted that for articles with missing data and information, we emailed the corresponding author and asked them to send us the necessary information. Finally, two authors extracted all data from eligible full-text documents through Excel.

### Synthesis of results

After the data were stored and managed in MS Excel for processing, to synthesize data, one author (KHM) checks all imported data (e.g., spell check, cell formatting). Then, descriptive statistics (frequency and percentage) were used to summarize the collected data. Descriptive data obtained from the findings of the included articles were organized into tables and figures based on themes to present the findings of this review, which guided the study aims by (KB, AH, and ASH).

### Ethical considerations

The protocol of this study was approved by the ethical committee of Kerman University of Medical Sciences) IR.KMU.REC.1400.606).

## Results

### Study selection process

From the 348 non-duplicate articles found using the search strategy, 55 articles were selected for inclusion (Fig. 1). Summarized findings from selected articles are included in Table 1.

### Critical appraisal of individual sources of evidence

The findings of the quality assessment of studies based on MMAT are presented in Appendix A.

### Results of the reviewed studies

Most of the studies were done in Italy (n = 19, 34.55%) [26–28, 31, 33, 36, 37, 43, 46–49, 57, 62, 63, 68, 76, 79]. After Italy, 14.24% and 8.9% of the studies were conducted in the USA (n = 8) [29, 42, 50, 51, 56, 60, 61, 72] and China (n = 5) [32, 54, 59, 65, 73], respectively. (More details in Table 1). Also, most of the studies were RCT (n = 26) [28–31, 35, 36, 40–42, 47, 48, 50, 53–55, 63, 66, 68, 70, 72, 73, 75, 76, 78–80].

**Fig. 2:**
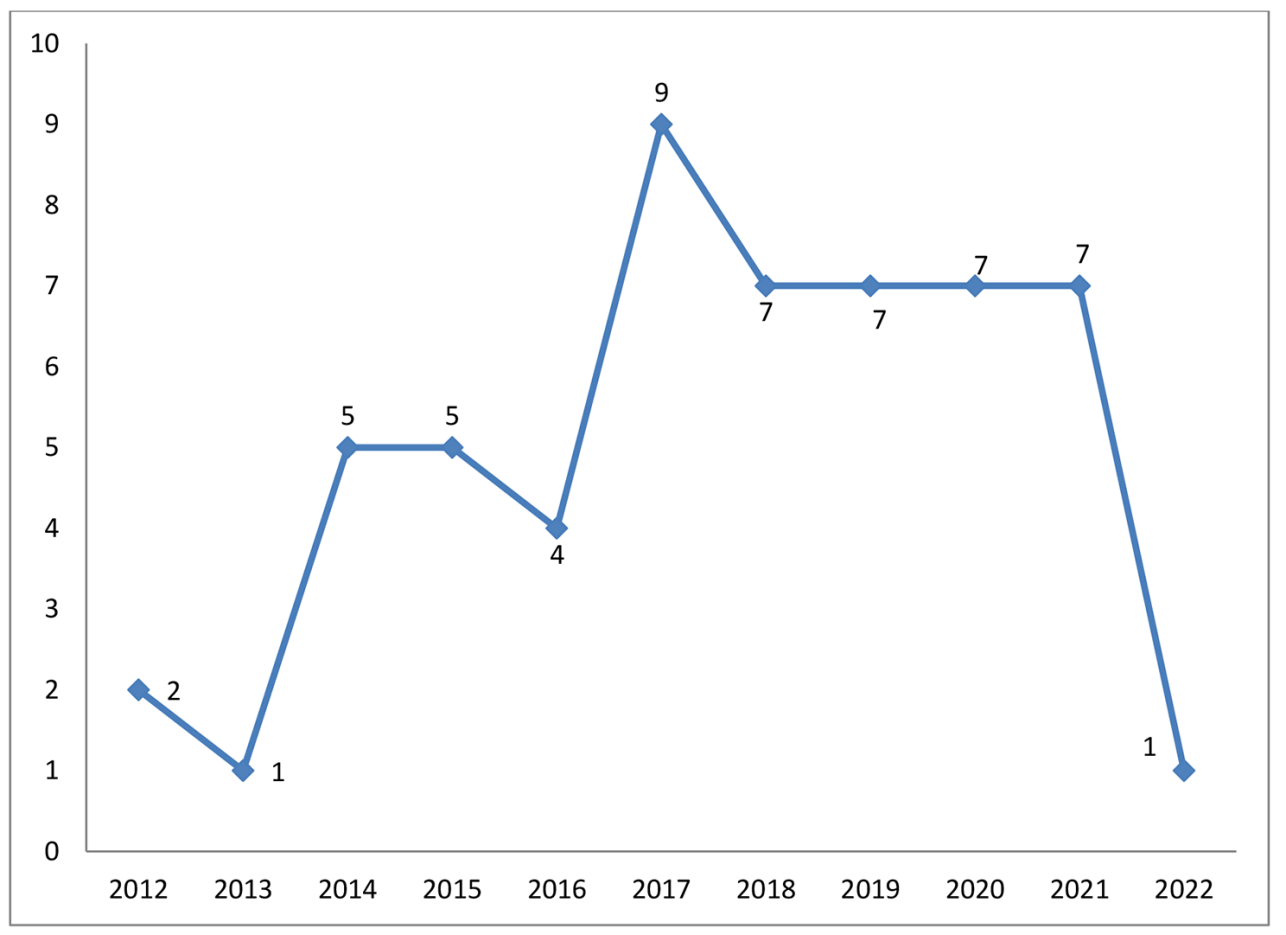
Distribution of the studies in terms of publication year

As shown in Fig. 2, most articles on robots for upper limb rehabilitation were published in 2017 (n = 9) [33–35, 60, 61, 67, 72, 79, 80], 2020 (n = 7) [46–50, 63, 64, 69], and 2021 (n = 7) [51–55, 65, 73]. (More details in Table 1)

**Fig. 3:**
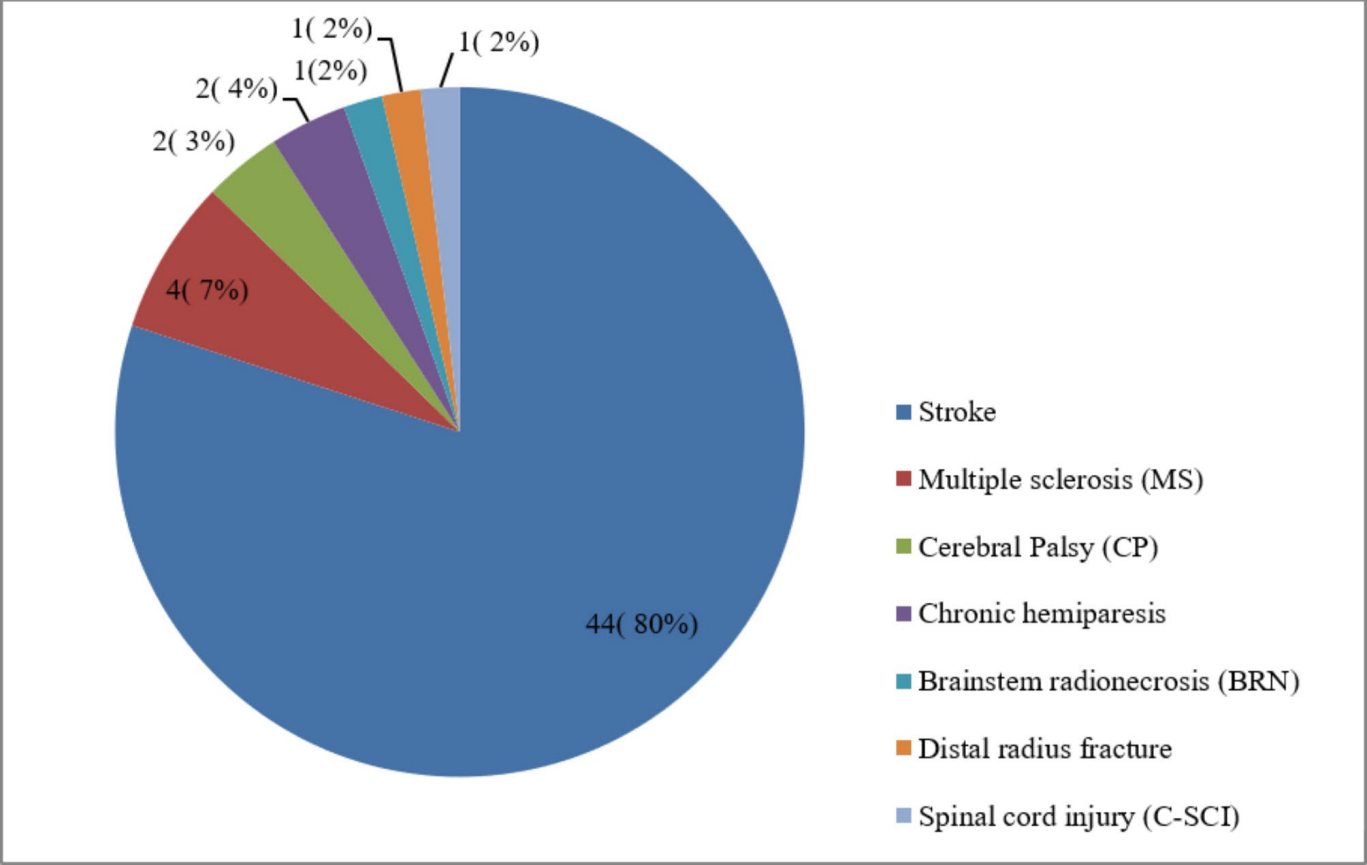
The distribution of the studies based on diseases and injuries leading to upper limb disabilities

Robots were used to rehabilitate seven categories of diseases or complications leading to upper limb disability (Fig. 3). Most robots were used for patients with stroke (n = 44, 80%) [28, 29, 31–35, 37–42, 44, 45, 48–62, 64–66, 68, 70, 72–80]. Other diseases with frequencies and percentages are shown in Fig. 3.

Eight different levels of upper limb disability were identified (Table 2). The highest frequency of disabilities was related to the entire upper limb (n = 30). Wrist (n = 10), fingers (n = 7) and arm (n = 7) were other parts of the upper limb that had the highest level of disabilities. (More details in Table 1)

Table 4 shows that some robots were designed to be used to rehabilitate the entire upper limb (n = 15). Then, robots were mostly used for wrist (n = 14), finger (n = 12), and shoulder (n = 11) rehabilitation, respectively. (More details in Appendix B)

**Use of telerehabilitation and virtual reality in robots.**

60.52% of the studies used games and virtual reality to rehabilitate the upper extremities in robots [26, 30–32, 36, 38, 40, 42–51, 54, 55, 57, 59–62, 64, 66–73, 75](n = 34). Moreover, 3.56% of studies had used telerehabilitation in their robots (n = 2) [32, 35].

### Evaluations in upper limb rehabilitation robots

The number of participants in the evaluation of upper limb rehabilitation robots varied from one [43, 46, 59, 64, 77] to 224 [48]. According to Tables 3, 89% of the studies used both males and females in the robot evaluation process(n = 49) [26–29, 31–42, 44, 45, 47–58, 60–63, 65–76, 78–80]. Most of the participants were over 18 years old (n = 47, 85%) [26–29, 31, 33–51, 53–60, 62, 63, 65–68, 70–73, 75, 78–80]. Moreover, in some studies, the age of the participants was less than or equal to 18 years (n = 4, 7%) [30, 67, 69, 76]. The minimum and maximum duration of evaluation processes were 30–45 min [26] to 12 months [52], respectively. Also, 83% of the studies included both male and female patients in the robot evaluation process. One study included only women [43], and two studies focused on men [46, 77] (More details in Appendix B).

The duration of treatment of patients in 38% of studies was less than one month (n = 21) [26, 28, 32, 33, 35, 39, 47, 49, 51, 54, 56, 58, 60, 62–64, 66, 68, 72, 73, 75]. In 41% of the studies (n = 23) [29–31, 34, 36, 40, 43–46, 50, 53, 55, 57, 61, 65, 67, 69, 71, 76, 78–80], the duration of treatment of patients was between one and two months. In other studies, the duration of treatment of patients was more than three months [27, 42, 48, 52, 70](n = 5, 9%) (More details in Appendix B).

According to Tables 5, 14 types of evaluation were performed for upper limb rehabilitation robots. “Evaluation and measurement of upper limb function and dexterity” was the most common type of evaluation (n = 52). “Range and motor skills and functional strength of the hand” were ranked next (n = 12). Then, “Neuropsychological assessment” was the most common type of evaluation (n = 11). As shown in Tables 5, 85 different evaluation methods were used. “Fugl-Meyer Upper Extremity score” was the most used evaluation method in studies (n = 30). Then, “Barthel Index (BI)” (n = 11), “Modified Ashworth Scale (MAS)” (n = 11), and “Box and Block Test (BBT)” (n = 11) were the most widely used evaluation methods.

### Outcomes of using upper limb rehabilitation robots

The most important outcomes of upper limb rehabilitation robots were “Improvement in musculoskeletal functions”, “No adverse effect on patients”, and “Safe and reliable treatment”, respectively. Other outcomes are listed in Table 6. (More details in Appendix B).

**Table 6:** Outcomes of using rehabilitation robots

Outcomes (references)	Outcomes frequency based on the number of references
Improvement in musculoskeletal functions (Musculoskeletal strength, sensation, perception, vibration, muscle coordination, less spasticity, flexibility and range of motion) [26–36, 38–51, 53–63, 65–70, 72–80]	51
No adverse effect on patients [36, 38, 46, 47, 64, 70, 76]	7
Safe and reliable treatment [31, 37, 47, 54, 68, 76]	6
The efficiency of rehabilitation robots [28, 32, 38, 68]	4
Effectiveness of rehabilitation robots [40, 54, 68, 79]	4
Reduced or relieved pain [48, 68, 75]	3
Increasing patients’ interest and motivation to perform rehabilitation exercises [30, 32, 71]	3
Rehabilitation robots’ feasibility for train of patients [71, 77, 79]	3
Increasing the patient’s independence in performing rehabilitation exercises [41, 46, 62]	3
Performing repetitive and long exercises very easily with the help of the robot [61]	1
Reducing the duration of rehabilitation exercises [62]	1
Increasing adherence to rehabilitation exercises and more participation in treatment processes [52]	1
Improving the quality of life [61]	1
Improving the quality of the rehabilitation process [64]	1

## Discussion

In this scoping review, outcomes of rehabilitation robots for upper limb disabilities and different methods for evaluating rehabilitation robots were identified. Most robots were used for the rehabilitation of patients with stroke. Along with robots, telerehabilitation and virtual reality, technologies were also used for upper limb disabilities rehabilitation. “Evaluation and measurement of upper limb function and dexterity” was the most common type of evaluation among the 14 types performed for upper limb rehabilitation robots. Also, we identified different outcomes of using rehabilitation robots for upper limb disabilities.

As discussed above, telerehabilitation and virtual reality were two types of technology used along with rehabilitation robots. Some studies [3, 82, 83] showed that if special interventions such as game-based virtual reality, and telerehabilitation with rehabilitation robots are used, upper limb function, mental health and patients’ participation in rehabilitation exercises can be improved. Moulaei et al. [3], showed in a scoping review that telerehabilitation could lead to “improving in musculoskeletal functions”, “increasing patients’ interest and motivation to perform rehabilitation exercises”, “increasing adherence to rehabilitation exercises and more participation in treatment processes”, “remote monitoring and control of patients”, “reducing or relieved pain” and “improving quality of life”. Fernández-Vázquez et al. [84], also pointed out that virtual reality as a very interesting tool in treating upper limb disorders along with rehabilitation robots can improve the motor function of the upper limb, increase users’ motivation and enjoyment, a large number of repetitions or high intensity rehabilitation. Repetitive and long exercises become easy and reduce the time needed to perform therapeutic exercises. Some studies [85–87] stated that for telerehabilitation systems and virtual reality to be as effective as robots, they should be easy to use, user-friendly, and have a beautiful and appropriate user interface. Zanatta et al. [87] pointed out that when telerehabilitation systems, robots and virtual reality are usable and attractive, patients’ motivation to perform rehabilitation exercises increases, their satisfaction with treatment processes increases, and efficiency and effectiveness increase. Therefore, to combine the robot with telerehabilitation or virtual reality to be effective, various factors such as usability, suitable user interface and even technical support should be considered in design, development and implementation.

The findings of this study and other reviews [2, 22, 88, 89] have shown that rehabilitation robots have been able to improve the musculoskeletal status of patients with upper limb disabilities. FMA-UE scores obtained from different studies showed that robot-based treatments can improve upper limb musculoskeletal function [1, 90–92]. In a review of the literature on therapeutic robotics in chronic stroke survivors and individuals with hemiparesis, the FMA total score improved from 2.1 to 15.1 and the ARAT improved to 11.1 [1, 90–92]. Milot et al. [91], tested 20 volunteers with mild to moderate chronic stroke to investigate the robot’s functional outcomes on the upper limb. Their study showed that training with the robot led to significant improvements in FMA, BBT, MAL, WMFT, and quantitative amounts of strength and speed, and these statuses were maintained in the 3-month follow-up [91]. Cimolin et al., [93] conducted a clinical trial using the Armeo Spring device on children aged 4 to 17 years with cerebral palsy. The subjects of this study showed promising improvements in different movement patterns and hand functions. It seems that to increase the effect of robots on improving upper limb disabilities, it is better to consider a number of factors. For example, the mechanical characteristics of rehabilitation robots may affect outcomes. Exoskeleton robots can control several joints simultaneously, leading to tight physical interaction between humans and robots, which may increase the patient burden.

Additionally, the exoskeleton robot’s high inertia to its complex structure can intervene with manipulation. Or, in the mechanical dimensions of the robot, the degree of freedom should be considered an important factor in the design. For example, intervention with six degrees of freedom in a three-dimensional space is very challenging for stroke patients with moderate to severe motor impairment [1]. The term “paradox of reduced number of degrees of freedom” states that to train a patient with a severe movement disorder, one should first use the lowest number of degrees of freedom, then gradually increase the number of degrees of freedom as improvements are achieved [94]. On the other hand, if the robots are not light, they can put pressure on different organs of patients during exercise. Also, rehabilitation robots can be costly, except for people who might profit from using them for training. As a result, efforts should be made to make rehabilitation robots as cost-effective as possible. Finally, these devices should be easily available to people, and awareness of them as an effective and helpful alternative treatment should also continuously increase.

Although, in this review we observed that rehabilitation robots have no adverse effect on patients, in some studies, the complications of rehabilitation robots have been mentioned [89]. Bessler et al. [95], believed that the safety of using rehabilitation robots in clinical trials should be confirmed for monitoring and reporting adverse events. In a systematic literature review, Bessler et al. [89], gathered information on the type of adverse events associated with the training of static robotic gait trainers. They identified about 17 adverse events per 100 people trained on a stationary robotic gait trainer. The most common types of side effects were classified into two categories: side effects related to soft tissue and musculoskeletal side effects. The third category included physiological adverse events (such as sudden changes in blood pressure) that seem to be unrelated to the mechanical settings of the robotic device in most cases, but are generally considered to be related to the activity engaged. Side effects associated to soft tissue in trainers also included things such as skin redness, skin irritation, open skin lesions and bruising, skin abrasion, as well as hurt and pain in soft tissue areas. Musculoskeletal side effects also included tendinopathy, muscle pain, tibial fracture, back and malleolus pain, and joint discomfort and pain [89]. Therefore, rehabilitation robots should be designed to minimize the side effects related to all three dimensions, i.e., soft tissue, musculoskeletal, and physiological. First of all, it can be said that the designers of these systems must ensure that there are no side effects before using robots by patients or therapists, so that they can be used safely for rehabilitation. Then, the therapists should set the rehabilitation exercises in terms of dosage and duration in such a way that the side effects caused by the low or high use of the robot do not occur for the patients.

Besides, we should be able to improve the design, development and evaluation of rehabilitation robots so that they have the necessary quality to provide rehabilitation services. Abu-Dakka et al. [96] believed that rehabilitation robots should be designed in a way that can be easily used by patients, therapists, and clinicians, increase the effectiveness of doctors’ treatments and make patients’ daily activities easier. To achieve these goals, rehabilitation robots must have some functional requirements, including: stability, safety, adaptability to patient needs, accept a wide range of patients, providing a full Range of Motion (ROM), being equipped with the necessary sensors for haptic and visual feedback, etc. [97, 98]. Also, in the design and evaluation of rehabilitation robots, we must consider the views and opinions of users (patients and therapists), because these people are the end users of these rehabilitation tools. Considering the perspectives and opinions of patients and therapists in the design process also makes the device develop according to the patients’ needs and preferences. As a result, their level of satisfaction and continued use of this device improves. Zanatta et al., [87] believed that considering the patient’s perspective when designing or evaluating a rehabilitation device is essential to ensure adequate engagement and adherence to treatment. Or when the usability of a rehabilitation robot is suitable for users, according to Nielsen [99], learnability, efficiency, memorability and satisfaction of users improve, while the rate of errors also decreases.

Accordingly, a patient who perceives a device as usable is likely also report a positive perception as a concern: ease of learning the system’s function and behavior, effort expended to reach a goal, ease of remembering the system’s performance for any further use, the system’s ability to easily support and recover the case of errors during use and the pleasant design of the system. Thus, assessing such aspects provides important insights into the acceptability and perceived usefulness of devices, thereby allowing an understanding of how to improve patient motivation during treatment. As emphasized in Monardo’s study [100], motivation plays an essential role during the rehabilitation process because it helps create a sense of patient competence and satisfaction. In addition, patient satisfaction with treatment-rehabilitation processes performed with the help of rehabilitation devices was associated with stronger treatment compliance [101]. As a result, both factors can be considered key factors for the effectiveness of treatment-rehabilitation processes. On the other hand, the anatomical structure of the upper limb, the severity and degree of the patients’ disabilities, and the age group of the patients (for example, children or the elderly) should also be considered when designing and developing a rehabilitation tool. For example, a rehabilitation robot may only be used by children and not by elderly persons [102] or a robot may be designed for finger rehabilitation-only [103].

According to the findings of the studies, we found that robots were a safe and reliable treatment for patients. Therefore, it is necessary to consider a set of factors for the rehabilitation robot to guarantee patient safety and maintain its reliability for patients. Sampson et al. [104], evaluated the BUiLT + VR system in hemiparetic patients with upper extremity disability and found that this system is reliable for treating patients and can be administered safely. They believed that through Rests between games, ramping of the time spent playing in the early sessions, and adjustments to seating, unit height and angle, it could overcome the challenges related to patient safety and increase its reliability for users. Other studies show that the designed rehabilitation robot should ensure the safety of the patients from the aspects of using the common sensor to monitor and manage the force information of the patients during movement, when the reaction force made by the muscle tension is very high. Also, when the patient experiences a muscle spasm, the robot should automatically stop the current movement so that the muscle is not strained. Therefore, to prevent repeated injury to patients, the space and working environment should be restricted by limiting the switch within a reasonable range. The rehabilitation robot’s speed of displacement and movement should also be limited by the software used to control the robot. The operator or therapists should be able to rationally adjust the parameters of the driving device, and control and monitor the movement status of the robot in real time. Pressing the stop button is very important to avoid accidents. Also, to avoid damage created by the rotation of the joints due to gravity, the rehabilitation robot’s ability to self-lock should be considered [105].

Reduction or relief of pain was another outcome of this review. Some studies [75, 95] have shown that robots can reduce or relieve pain in patients, but factors must be considered when designing or using robots so that the robot can reduce pain more effectively. For example, exceeding forces on the human musculoskeletal system, at the same time misalignment will make high pressure and shear forces via slipping at the cuffs or straps [106, 107]. Therefore, forces that are not considered or compensated for in the design of the robot or its interface are easily transmitted to the musculoskeletal systems. On the other hand, if the torques and forces are too high or act in arbitrary directions on the musculoskeletal system, they can create an additional load, resulting in pain and damage to the bones, joints, and muscles [95, 106]. Therefore, when designing robots, pain thresholds should be considered for pressures and forces applied during accidental contact such as collisions or closing situations with a robot [108]. Different limit values for many different parts of the human body are accepted by ISO/TS 15,066 [95]. The patient’s position to perform therapeutic exercises with the help of the robot is also very effective in reducing pain. The robot developed in Kim et al.‘s [75] study allows patients to be comfortable and supported in a supine position. This way can easily be linked to other pain therapeutic methods, such as interferential current therapy or hot pack application. For example, installing a friction plate to forbid the compensation of the scapula and to increase the therapy’s effectiveness and adjust the robotic arm’s height using a linear actuator can be very helpful [75].

Duration and intensity of the therapy through the robot are two other factors that effect on the robots in motor control and improving pains. By examining the effectiveness of robots on function and structure in patients with limited upper limb function, Ferreira et al., [109] showed that when robot-assisted therapy is used with conventional treatment at the same dose and duration, robot-assisted therapy has a significant effect motor control. The group’s analysis also proposed an effect of the number of sessions and duration of treatment on some estimated impacts. The greater number of sessions and the volume of treatment affect motor control [109]. The more significant treatment dose effect was proposed by Lohse et al. [110]; however, time as a representation of dose is a rather rough estimate and supplies no evidence of the actual amount of movement or types of movement, nor does it account for periods of inactivity or rest [111]. In this respect, a previous study showed that, although there is no agreement, the minimum dose should be at least 16 h of exercise [112]. Poor methodological quality and lower dose and duration of treatment may negatively affect the estimated effects. So, therapists usually consider this approach because it has very few or no side effects [109]. Moreover, some studies have shown that low or high intensity exercise therapy can increase the risk of injury and worsening pain in the hemiplegic shoulder [113]. In the study by Kim et al. [75], participants in the intervention or control groups performed stretching exercises for 30 min per day, five times per week for four weeks; Shoulder pain in the intervention group (they used the robot) was worse than the control group. The authors of this research concluded that this stretching exercise was performed for patients with excessive intensity and more than 30 min of stretching exercise caused pain in some patients even when it was performed with low intensity.

As our study showed, robots can increase patients’ interest and motivation to perform rehabilitation exercises. Therefore, one of the other factors that can be considered to increase patients’ use of robots and their interest and motivation to perform rehabilitation exercises is the use of games and virtual reality. Creating a degree of entertainment through games and virtual reality can improve patients’ adherence to rehabilitation exercises and increase patients’ attention span to spend more time on their rehabilitation program [114]. Kafri et al. [115], investigated the effect of virtual reality on patients after a stroke. Patients showed an improvement in activity and reported that they enjoyed the game. Virtual reality also supplies direct visual feedback and can empower patients with a sense and awareness of control over their recovery. Consequently, it is imaginable that people who train with virtual reality and games will enhance their performance when interacting with an avatar presented on the screen [114]. In another study, Gomez et al., [116] evaluated patients with multiple sclerosis using games, in which the experimental group received both game sessions and conventional treatment. This study showed that after using the games, interest and motivation to perform rehabilitation exercises improved in the patients.

Efficiency and effectiveness of rehabilitation robots, increasing the patient’s independence in performing rehabilitation exercises, rehabilitation robots’ feasibility for training patients, performing repetitive and long exercises very easily with the help of the robot, reducing the duration of rehabilitation exercises, increasing adherence to rehabilitation exercises and more participation in treatment processes, and improving quality of life and rehabilitation process were other outcomes related to rehabilitation robots identified in this study. But it must be said that these outcomes or other mentioned outcomes will not be created spontaneously due to the use of rehabilitation robots, but a series of main factors or capabilities must be included in the robots so that these outcomes can be created and have a and beneficial effect in rehabilitation. For example, Laut et al. [117] believed that rehabilitation robots could potentially increase efficiency and access to treatment by having capabilities such as providing continuous training for a long period of time and collecting data to evaluate progress. Furthermore, by pairing rehabilitation robots with health information technologies and presenting assessment and performance data all over the internet to the therapist, different treatment processes can be moved out of specific facilities and into patients’ homes with remote management by a therapist. This can let a therapist treat several patients simultaneously, greatly enhancing the numbers of patients treated and the efficiency of the rehabilitation robot and therapist.

In other words, changing from a hospital to a home rehabilitation program can reduce costs while making rehabilitation more accessible [117]. Francisco et al. [118] also stated that by providing frequent and intensive training through rehabilitation robotics, the effectiveness of conventional occupational therapy or physiotherapy can be improved by providing more coherent, accurate, and precise treatment. Therefore, it should be said that for robots to be able to teach patients better or be more efficient, they must be able to provide frequent and intensive training. Also, in order to improve the efficiency and effectiveness of a rehabilitation device, the satisfaction of patients should be maximized. According to Tousignant et al.‘s study [119], satisfaction is an important indicator and factor for the degree of efficiency and effectiveness. Its high level can increase patient motivation, and adherence to treatment, and improve compliance with treatment. Also, it should be noted that satisfaction is one of the important factors in the quality of health care; it can affect adherence to treatment plans, improve clinical outcomes [120], and motivation of patients to perform rehabilitation exercises [121]. In order to maximize satisfaction, the robot must be designed according to the needs and preferences of its users, be usable and easy to use, and have no side effects [122, 123].

In addition, concerning adherence to rehabilitation exercises, it can be said that considering technologies such as sensors and computer game technologies for home rehabilitation or mRehab (mobile rehabilitation) systems in robots greatly increases the possibility of objective quantification of adherence [124]. Also, for patients to engage and be challenged while performing rehabilitation exercises at home in the physical absence of the therapist, there is a need to carefully design scenarios and motivational features that guarantee the patient’s adherence to the treatment plan [125]. Increasing the quality of life was another important outcome of using rehabilitation robots identified in our study. In the study by Kutner et al. [126], it was shown that patients who had suffered a stroke experienced a faster increase in quality of life (in physical, mental and social dimensions) after participating in targeted interventions that focused on improving upper limb strength. Mohammed et al. [127], in their study of patients with upper and lower limb amputation, defined the quality of life in three dimensions physical, mental and social health. Therefore, it should be said that when a rehabilitation robot is designed, in order to increase the quality of life of the patients, it should be focused on all three dimensions of the patients’ health, physical, psychological and social.

In general, it can be said that although the findings of the present study showed that rehabilitation robots could lead to positive outcomes for people with upper limb disabilities with various diseases or injuries, but if the factors that lead to these outcomes in If these robots are not considered, they will never have the necessary efficiency, effectiveness and quality.

### Prospects of rehabilitation robots

It is predictable that rehabilitation robots will be applied much more than before in an era where the cost of manual therapy is becoming more expensive. To better popularize and commercialize this goal, future development needs to increase the universality of rehabilitation robots, focusing on portable, lightweight, reconfigurable, smart, and equipment based on artificial intelligence and machine learning techniques, new methods of treatment and thinking to the field of rehabilitation under the hypothesis of guaranteeing safety, mass production and development in a cheap direction [24]. For example, machine learning techniques may ,allow a robot to independently adapt to the changing needs of each customer over time and perform specific tasks, such as autonomous gaming [128]. Another example is the portability of the robot, which allows users to carry the robot system for deployment inside homes or medical centers [129]. People can use portable rehabilitation robots not only at home and in the treatment center, but also in nature and parks. On the other hand, Smart rehabilitation is the future development trend. It should be broken through the human-machine interface barrier, develop multidisciplinary and interdisciplinary collaboration and communication, improve and strengthen cooperation with rehabilitation medicine and artificial intelligence, actively participate in the design, development and evaluation of rehabilitation robots, and improve rehabilitation robots for providing better and more quality service to people.

Moreover, in the future, there should be more focus on “human nature” in the design and development of rehabilitation robots. Human nature means that robots have capabilities such as “perception, thinking, action and cooperation, expression and communication, and learning and adaptability and complete training and operations independently or with the assistance of an automatic machine” [24]. At present, rehabilitation robots cannot perform all the tasks of professional rehabilitation personnel; especially, the ability of self-learning, adaptability, flexibility and creativity of robots needs many theoretical developments. The most challenging and most difficult thing for rehabilitation robots is the benevolence and moral sense of medical staff [130]. Since in the future software packages and powerful hardware equipment will be produced, many of these problems can be easily solved.

### Limitations of the study

There are a few limitations in this study. In the present study, only articles in English were reviewed; it is better to include articles published in non-English languages in future studies. Also, to find related studies, we searched four databases, Scopus, IEEE, PubMed, and Web of Science. It is suggested that more studies be done in more databases to obtain more comprehensive results.

## Conclusion

This scoping review indicates that upper limb rehabilitation robots can improve musculoskeletal functions (Musculoskeletal strength, sensation, perception, vibration, muscle coordination, less spasticity, flexibility, and range of motion); avoid serious side effects or adverse effects on the patient, provide safe and reliable treatment, reduce pain, increase the patient’s independence in performing rehabilitation exercises, reduce the duration of rehabilitation exercises, increase adherence to rehabilitation exercises and treatment processes, and quality of life and rehabilitation processes. The use of rehabilitation robots is growing strongly, especially in developed countries, and it seems that this new rehabilitation technology has had significant effects in helping to improve upper limb disabilities.

Also, rehabilitation robots could provide a platform for motivating people with upper limb disabilities to carry out more rehabilitation exercises without a therapist, which could maximize recovery.

## Electronic supplementary material

Below is the link to the electronic supplementary material.

Supplementary Material 1 Aims and scope statement

Supplementary Material 2: Appendix A

Supplementary Material 3: Appendix B

Supplementary Material 4: Appendix C

## Data Availability

The datasets used and analyzed during the current study are available from the corresponding author on reasonable request.

## Abbreviations

9HPT: Nine-Hole Peg Test
AHA: Assisting Hand Assessment
AHF: Arm-Hand Function
AMAT: Arm Motor Ability Test
ARAT: Action Research Arm Test
BBT: Box and Block Test
BI: Barthel Index
BIT: Behavioral Inattention Test
BRN: Brainstem radionecrosis
C-SCI: Spinal cord injury
CBS: Catherine Bergego Scale
CEQ: Credibility/Expectancy Questionnaire
CP: Cerebral Palsy
DAS: Disability Assessment Scale
DASH: Disabilities of Arm, Shoulder, and Hand
DN4: Douleur Neuropathique 4
DSEMG: Dynamic Surface Electromyography
Euro-QOl: Quality of Life
FIM: Functional Indipendence Measure
FMA-UE: Fugl-Meyer Upper Extremity score
FMRI: Functional magnetic resonance imaging
fNIRS: Functional near infrared spectroscopy
GP: Grooved Pegboard Test
IMI: Intrinsic Motivation Inventory
JTHFT: Jebsen-Taylor Hand Function Test
K-SDQ: Shoulder Disability Questionnaire
MAL: Motor Activity Log
MAS: Modified Ashworth Scale
MAS: Modified Ashworth Scale
MCS: Mental health Composite Score
MeSH: Medical Subject Heading
MFT: Manual Function Test
MI: Motricity Index
MMSE: Mini-Mental State Examination
MRI: Magnetic Resonance Imaging
MRS: Modified Rankin Scale
MS: Multiple sclerosis
MSQOL-54: Multiple Sclerosis Quality of Life-54
NEADL: Nottingham Extended Activities of Daily Living
NIHSS: National Institutes of Health Stroke Scale
NRPS: Numeric Rating Pain Scale
NRS: Numerical Rating Scale
OCS: Oxford Cognitive Screen
PCS: Physical health Composite Score
PEDI: Pediatric Evaluation of Disability Inventory
POMA: Performance Oriented Mobility Assessment
PRWHE: Patient-Rated Wrist and Hand Evaluation
QOM: Quality of Movement
QUEST: Quality of Upper Extremity Skills Test
Quick-DASH: Quick version of the Disabilities of the Arm, Shoulder, and Hand
RCI: Reliable Change Index
RCT: Randomized controlled trials
ROCF: Rey Osterrieth complex figure test
ROM: Active or Passive Range Of Motion
SCWT: Stroop Color and Word Test
SDMT: Symbol Digit Modalities Test
SDMT: Symbol Digit Modalities Test
SDS: Self-Depression Scale
SF-36: Short Form Health Survey
SIS: Stroke Impact Scale
SIS: Stroke Impact Scale
TAM: Total active mobility
TMT: Trail making test
TSS: Tremor Severity Scale
UL: Upper limb
USE: Usefulness, Satisfaction and Ease-of-use
VAS: Visual analog scale
VAS: Visual analogue scale
WHODAS: World Health Organization Disability Assessment Schedule
WMFT: Wolf Motor Function

## References

[1] Lee SH, Park G, Cho DY, Kim HY, Lee J-Y, Kim S, Park S-B, Shin J-H (2020). Comparisons between end-effector and exoskeleton rehabilitation robots regarding upper extremity function among chronic stroke patients with moderate-to-severe upper limb impairment. Sci Rep.

[2] Vélez-Guerrero MA, Callejas-Cuervo M, Mazzoleni S (2021). Artificial Intelligence-Based Wearable Robotic Exoskeletons for Upper Limb Rehabilitation: a review. Sens (Basel).

[3] Moulaei K, Sheikhtaheri A, Nezhad MS, Haghdoost A, Gheysari M, Bahaadinbeigy K (2022). Telerehabilitation for upper limb disabilities: a scoping review on functions, outcomes, and evaluation methods. Archives of Public Health.

[4] Poltawski L, Allison R, Briscoe S, Freeman J, Kilbride C, Neal D, Turton AJ, Dean SJD. rehabilitation: Assessing the impact of upper limb disability following stroke: a qualitative enquiry using internet-based personal accounts of stroke survivors. 2016, 38(10):945–51. 10.3109/09638288.2015.1068383 PMC4819828 26200448

[5] Maciejasz P, Eschweiler J, Gerlach-Hahn K, Jansen-Troy A, Leonhardt S (2014). A survey on robotic devices for upper limb rehabilitation. J Neuroeng Rehabil.

[6] Vélez-Guerrero MA, Callejas-Cuervo M, Mazzoleni S (2021). Design, Development, and testing of an Intelligent Wearable Robotic Exoskeleton Prototype for Upper Limb Rehabilitation. Sens (Basel).

[7] Wu Q, Wu H (2018). Development, dynamic modeling, and Multi-Modal Control of a therapeutic exoskeleton for Upper Limb Rehabilitation Training. Sens (Basel).

[8] Qassim HM, Wan Hasan WZ. A Review on Upper Limb Rehabilitation Robots. Appl Sci 2020, 10(19).

[9] Richards L, Hanson C, Wellborn M, Sethi A (2008). Driving motor recovery after stroke. Top Stroke Rehabil.

[10] Qian Q, Nam C, Guo Z, Huang Y, Hu X, Ng SC, Zheng Y, Poon W (2019). Distal versus proximal - an investigation on different supportive strategies by robots for upper limb rehabilitation after stroke: a randomized controlled trial. J Neuroeng Rehabil.

[11] Langhorne P, Bernhardt J, Kwakkel GJTL. Stroke rehabilitation. 2011, 377(9778):1693–1702. 10.1016/S0140-6736(11)60325-5 21571152

[12] Qian Z, Bi ZJAiME. Recent development of rehabilitation robots. 2015, 7(2):563062.

[13] Schweighofer N, Choi Y, Winstein C. Gordon JJAJoPM, Rehabilitation: Task-oriented rehabilitation robotics. 2012, 91(11):S270-S279. 10.1097/PHM.0b013e31826bcd42 23080042

[14] Ennaiem F, Chaker A, Laribi MA, Sandoval J, Bennour S, Mlika A, Romdhane L, Zeghloul SJAS. Task-Based design approach: development of a planar cable-driven parallel robot for upper limb rehabilitation. 2021, 11(12):5635.

[15] Johnson MJ, Loureiro RC, Harwin WSJISR. Collaborative tele-rehabilitation and robot-mediated therapy for stroke rehabilitation at home or clinic. 2008, 1(2):109–21.

[16] Nizamis K, Athanasiou A, Almpani S, Dimitrousis C, Astaras AJS. Converging robotic technologies in targeted neural rehabilitation: A review of emerging solutions and challenges. 2021, 21(6):2084. 10.3390/s21062084 PMC8002299 33809721

[17] Wege A, Zimmermann A. Electromyography sensor based control for a hand exoskeleton. In: *2007 IEEE International Conference on Robotics and Biomimetics (ROBIO)*: 2007: IEEE; 2007: 1470–1475.

[18] Wang Q, Markopoulos P, Yu B, Chen W, Timmermans A (2017). Interactive wearable systems for upper body rehabilitation: a systematic review. J Neuroeng Rehabil.

[19] Herrera-Luna I, Rechy-Ramirez EJ, Rios-Figueroa HV, Marin-Hernandez AJISJ. Sensor fusion used in applications for hand rehabilitation: A systematic review. 2019, 19(10):3581–3592.

[20] Koutsiana E, Ladakis I, Fotopoulos D, Chytas A, Kilintzis V, Chouvarda I (2020). Serious Gaming Technology in Upper Extremity Rehabilitation: scoping review. JMIR Serious Games.

[21] Parker J, Powell L, Mawson S (2020). Effectiveness of Upper Limb Wearable Technology for improving activity and participation in adult stroke survivors: systematic review. J Med Internet Res.

[22] Kwakkel G, Kollen BJ, Krebs HIJN. repair n: Effects of robot-assisted therapy on upper limb recovery after stroke: a systematic review. 2008, 22(2):111–21. 10.1177/1545968307305457 PMC2730506 17876068

[23] PRISMA for Scoping Reviews. [https://prisma-statement.org/Extensions/ScopingReviews].

[24] Xue X, Yang X, Deng Z, Tu H, Kong D, Li N, Xu F (2021). Global Trends and hotspots in Research on Rehabilitation Robots: a bibliometric analysis from 2010 to 2020. Front Public Health.

[25] Hong QN, Pluye P, Fàbregues S, Bartlett G, Boardman F, Cargo M, Dagenais P, Gagnon M-P, Griffiths F. Nicolau BJRoc: Mixed methods appraisal tool (MMAT), version 2018. 2018, 1148552(10). 10.1016/j.jclinepi.2019.03.008 30905698

[26] Carpinella I, Cattaneo D, Bertoni R, Ferrarin M (2012). Robot training of upper limb in multiple sclerosis: comparing protocols with or without manipulative task components. IEEE Trans neural Syst rehabilitation engineering: publication IEEE Eng Med Biology Soc.

[27] Sale P, Bovolenta F, Agosti M, Clerici P, Franceschini M (2014). Short-term and long-term outcomes of serial robotic training for improving upper limb function in chronic stroke. Int J rehabilitation Res Int Z fur Rehabilitationsforschung Revue Int de recherches de readaptation.

[28] Pennati GV, Da Re C, Messineo I, Bonaiuti D (2015). How could robotic training and botolinum toxin be combined in chronic post stroke upper limb spasticity? A pilot study. Eur J Phys Rehabil Med.

[29] McCabe J, Monkiewicz M, Holcomb J, Pundik S, Daly JJ (2015). Comparison of robotics, functional electrical stimulation, and motor learning methods for treatment of persistent upper extremity dysfunction after stroke: a randomized controlled trial. Arch Phys Med Rehabil.

[30] Gilliaux M, Renders A, Dispa D, Holvoet D, Sapin J, Dehez B, Detrembleur C, Lejeune TM, Stoquart G (2015). Upper Limb Robot-Assisted therapy in cerebral palsy: a single-blind randomized controlled trial. Neurorehabilit Neural Repair.

[31] Taveggia G, Borboni A, Salvi L, Mulé C, Fogliaresi S, Villafañe JH, Casale R (2016). Efficacy of robot-assisted rehabilitation for the functional recovery of the upper limb in post-stroke patients: a randomized controlled study. Eur J Phys Rehabil Med.

[32] Song AG, Wu CC, Ni DJ, Li HJ, Qin HY (2016). One-therapist to three-patient Telerehabilitation Robot System for the Upper Limb after Stroke. Int J Social Robot.

[33] Trujillo P, Mastropietro A, Scano A, Chiavenna A, Mrakic-Sposta S, Caimmi M, Molteni F, Rizzo G (2017). Quantitative EEG for Predicting Upper Limb Motor Recovery in Chronic Stroke Robot-Assisted Rehabilitation. IEEE Trans Neural Syst Rehabil Eng.

[34] Saita K, Morishita T, Hyakutake K, Fukuda H, Shiota E, Sankai Y, Inoue T (2017). Combined therapy using botulinum toxin A and single joint hybrid assistive limb for upper-limb disability due to spastic hemiplegia. J Neurol Sci.

[35] Hsieh YW, Wu CY, Wang WE, Lin KC, Chang KC, Chen CC, Liu CT (2017). Bilateral robotic priming before task-oriented approach in subacute stroke rehabilitation: a pilot randomized controlled trial. Clin Rehabil.

[36] Gandolfi M, Vale N, Dimitrova EK, Mazzoleni S, Battini E, Benedetti MD, Gajofatto A, Ferraro F, Castelli M, Camin M et al. Effects of High-intensity Robot-assisted Hand Training on Upper Limb Recovery and Muscle Activity in Individuals With Multiple Sclerosis: A Randomized, Controlled, Single-Blinded Trial. Front Neurol 2018, 9. 10.3389/fneur.2018.00905 PMC6207593 30405526

[37] Germanotta M, Cruciani A, Pecchioli C, Loreti S, Spedicato A, Meotti M, Mosca R, Speranza G, Cecchi F, Giannarelli G et al. Reliability, validity and discriminant ability of the instrumental indices provided by a novel planar robotic device for upper limb rehabilitation. J Neuroeng Rehabil 2018, 15. 10.1186/s12984-018-0385-8 PMC5956822 29769127

[38] Kim GW, Won YH, Seo JH, Ko MH, EFFECTS OF NEWLY DEVELOPED COMPACT ROBOT-AIDED UPPER EXTREMITY TRAINING SYSTEMS (NEURO-X (2018). (R)) IN PATIENTS WITH STROKE: A PILOT STUDY. J Rehabil Med.

[39] Iwamoto Y, Imura T, Suzukawa T, Fukuyama H, Ishii T, Taki S, Imada N, Shibukawa M, Inagawa T, Araki H (2019). Combination of Exoskeletal Upper Limb Robot and Occupational Therapy improve activities of Daily living function in Acute Stroke Patients. J stroke Cerebrovasc diseases: official J Natl Stroke Association.

[40] Dehem S, Gilliaux M, Stoquart G, Detrembleur C, Jacquemin G, Palumbo S, Frederick A, Lejeune T (2019). Effectiveness of upper-limb robotic-assisted therapy in the early rehabilitation phase after stroke: a single-blind, randomised, controlled trial. Annals of Physical and Rehabilitation Medicine.

[41] Hung CS, Lin KC, Chang WY, Huang WC, Chang YJ, Chen CL, Grace Yao K, Lee YY (2019). Unilateral vs bilateral hybrid approaches for Upper Limb Rehabilitation in Chronic Stroke: a Randomized Controlled Trial. Arch Phys Med Rehabil.

[42] Conroy SS, Wittenberg GF, Krebs HI, Zhan M, Bever CT, Whitall J (2019). Robot-Assisted arm training in chronic stroke: addition of transition-to-Task Practice. Neurorehabilit Neural Repair.

[43] Bonanno L, Russo M, Bramanti A, Calabrò RS, Marino S (2019). Functional connectivity in multiple sclerosis after robotic rehabilitative treatment: a case report. Medicine.

[44] Leem MJ, Kim GS, Kim KH, Yi TI, Moon HI (2019). Predictors of functional and motor outcomes following upper limb robot-assisted therapy after stroke. Int J rehabilitation Res Int Z fur Rehabilitationsforschung Revue Int de recherches de readaptation.

[45] Kim DH, Kim KH, Lee SM (2020). The effects of virtual reality training with Upper Limb sensory Exercise Stimulation on the AROM of Upper Limb joints, function, and concentration in chronic stroke patients. Phys Medizin Rehabilitationsmedizin Kurortmedizin.

[46] Tartamella F, Chillura A, Pisano MF, Cacioppo A, Licari S, Caradonna D, Portaro S, Calabr RS, Bramanti P, Naro A. A case report on intensive, robot-assisted rehabilitation program for brainstem radionecrosis. Medicine 2020, 99(10). 10.1097/MD.0000000000019517 PMC7478746 32150113

[47] Solaro C, Cattaneo D, Basteris A, Carpinella I, De Luca A, Mueller M, Bertoni R, Ferrarin M, Sanguineti V. Haptic vs sensorimotor training in the treatment of upper limb dysfunction in multiple sclerosis: A multi-center, randomised controlled trial. J Neurol Sci 2020, 412. 10.1016/j.jns.2020.116743 32145522

[48] Aprile I, Germanotta M, Cruciani A, Pecchioli C, Loreti S, Papadopoulou D, Montesano A, Galeri S, Diverio M, Falsini C et al. Poststroke shoulder pain in subacute patients and its correlation with upper limb recovery after robotic or conventional treatment: A secondary analysis of a multicenter randomized controlled trial. Int J stroke: official J Int Stroke Soc 2020:1747493020937192. 10.1177/1747493020937192 32640881

[49] Aprile I, Guardati G, Cipollini V, Papadopoulou D, Mastrorosa A, Castelli L, Monteleone S, Redolfi A, Galeri S, Germanotta M. Robotic Rehabilitation: An Opportunity to Improve Cognitive Functions in Subjects With Stroke. An Explorative Study. Front Neurol 2020, 11. 10.3389/fneur.2020.588285 PMC7710798 33329334

[50] Kim GJ, Chen P (2020). Role of instruction adherence during highly structured robotic arm training on Motor Outcomes for individuals after chronic stroke. Am J Phys Med Rehabil.

[51] Bui KD, Wamsley CA, Shofer FS, Kolson DL, Johnson MJ (2021). Robot-Based Assessment of HIV-Related Motor and Cognitive Impairment for Neurorehabilitation. IEEE Trans Neural Syst Rehabil Eng.

[52] Flynn N, Froude E, Cooke D, Dennis J, Kuys S. The sustainability of upper limb robotic therapy for stroke survivors in an inpatient rehabilitation setting. Disabil Rehabil 2021. 10.1080/09638288.2021.1998664 34904486

[53] Terranova TT, Simis M, Santos ACA, Alfieri FM, Imamura M, Fregni F, Battistella LR. Robot-Assisted Therapy and Constraint-Induced Movement Therapy for Motor Recovery in Stroke: Results From a Randomized Clinical Trial. Front Neurorobotics 2021, 15. 10.3389/fnbot.2021.684019 PMC8335542 34366819

[54] Qu QM, Lin YN, He ZJ, Fu JH, Zou F, Jiang ZW, Guo FX, Jia J. The Effect of Applying Robot-Assisted Task-Oriented Training Using Human-Robot Collaborative Interaction Force Control Technology on Upper Limb Function in Stroke Patients: Preliminary Findings. *BioMed research international* 2021, 2021. 10.1155/2021/9916492 PMC8342143 34368358

[55] Abd El-Kafy EM, Alshehri MA, El-Fiky AAR, Guermazi MA, Mahmoud HM. The Effect of Robot-Mediated Virtual Reality Gaming on Upper Limb Spasticity Poststroke: A Randomized-Controlled Trial. *Games for health journal*. 10.1089/g4h.2021.0197 35100025

[56] Hu XL, Tong KY, Wei XJ, Rong W, Susanto EA, Ho SK (2013). The effects of post-stroke upper-limb training with an electromyography (EMG)-driven hand robot. J Electromyogr Kinesiol.

[57] Squeri V, Masia L, Giannoni P, Sandini G, Morasso P (2014). Wrist rehabilitation in chronic stroke patients by means of adaptive, progressive robot-aided therapy. IEEE Trans neural Syst rehabilitation engineering: publication IEEE Eng Med Biology Soc.

[58] Hsieh YW, Lin KC, Wu CY, Lien HY, Chen JL, Chen CC, Chang WH (2014). Predicting clinically significant changes in motor and functional outcomes after robot-assisted stroke rehabilitation. Arch Phys Med Rehabil.

[59] Chen W, Cui X, Zhang J, Wang J (2015). A cable-driven wrist robotic rehabilitator using a novel torque-field controller for human motion training. Rev Sci Instrum.

[60] McKenzie A, Dodakian L, See J, Le V, Quinlan EB, Bridgford C, Head D, Han VL, Cramer SC (2017). Validity of Robot-Based assessments of Upper extremity function. Arch Phys Med Rehabil.

[61] Housley SN, Wu D, Richards K, Belagaje S, Ghovanloo M, Butler AJ. Improving Upper Extremity Function and Quality of Life with a Tongue Driven Exoskeleton: A Pilot Study Quantifying Stroke Rehabilitation. Stroke Res Treat 2017, 2017. 10.1155/2017/3603860 PMC5748322 29403672

[62] Palermo E, Hayes DR, Russo EF, Calabro RS, Pacilli A, Filoni S. Translational effects of robot-mediated therapy in subacute stroke patients: an experimental evaluation of upper limb motor recovery. Peerj 2018, 6. 10.7717/peerj.5544 PMC6128258 30202655

[63] Picelli A, Munari D, Modenese A, Filippetti M, Saggioro G, Gandolfi M, Corain M, Smania N (2020). Robot-assisted arm training for treating adult patients with distal radius fracture: a proof-of-concept pilot study. Eur J Phys Rehabil Med.

[64] Bouteraa Y, Ben Abdallah I, Elmogy A (2020). Design and control of an exoskeleton robot with EMG-driven electrical stimulation for upper limb rehabilitation. Industrial Robot-the International Journal of Robotics Research and Application.

[65] Shi XQ, Heung HL, Tang ZQ, Li Z, Tong KY (2021). Effects of a Soft Robotic Hand for Hand Rehabilitation in Chronic Stroke Survivors. J stroke Cerebrovasc diseases: official J Natl Stroke Association.

[66] Hwang CH, Seong JW, Son DS (2012). Individual finger synchronized robot-assisted hand rehabilitation in subacute to chronic stroke: a prospective randomized clinical trial of efficacy. Clin Rehabil.

[67] Bishop L, Gordon AM, Kim H (2017). Hand Robotic Therapy in Children with Hemiparesis: a pilot study. Am J Phys Med Rehabil.

[68] Villafañe JH, Taveggia G, Galeri S, Bissolotti L, Mullè C, Imperio G, Valdes K, Borboni A, Negrini S (2018). Efficacy of short-term Robot-Assisted Rehabilitation in patients with Hand Paralysis after Stroke: a Randomized Clinical Trial. Hand (New York NY).

[69] Kuo FL, Lee HC, Hsiao HY, Lin JC (2020). Robotic-assisted hand therapy for improvement of hand function in children with cerebral palsy: a case series study. Eur J Phys Rehabil Med.

[70] Klamroth-Marganska V, Blanco J, Campen K, Curt A, Dietz V, Ettlin T, Felder M, Fellinghauer B, Guidali M, Kollmar A (2014). Three-dimensional, task-specific robot therapy of the arm after stroke: a multicentre, parallel-group randomised trial. Lancet Neurol.

[71] Vanmulken D, Spooren AIF, Bongers HMH, Seelen HAM (2015). Robot-assisted task-oriented upper extremity skill training in cervical spinal cord injury: a feasibility study. Spinal Cord.

[72] Kim GJ, Hinojosa J, Rao AK, Batavia M, O’Dell MW. Randomized Trial on the Effects of Attentional Focus on Motor Training of the Upper Extremity Using Robotics With Individuals After Chronic Stroke. Arch Phys Med Rehabil 2017, 98(10):1924–31. 10.1016/j.apmr.2017.06.005 28652064

[73] Chen ZJ, Gu MH, He C, Xiong CH, Xu J, Huang XL. Robot-Assisted Arm Training in Stroke Individuals With Unilateral Spatial Neglect: A Pilot Study. Front Neurol 2021, 12. 10.3389/fneur.2021.691444 PMC8297561 34305798

[74] Lee KS, Park JH, Beom J, Park HS. Design and Evaluation of Passive Shoulder Joint Tracking Module for Upper-Limb Rehabilitation Robots. Front Neurorobotics 2018, 12. 10.3389/fnbot.2018.00038 PMC6072863 30100871

[75] Kim MS, Kim SH, Noh SE, Bang HJ, Lee KM (2019). Robotic-assisted Shoulder Rehabilitation Therapy effectively improved Poststroke Hemiplegic Shoulder Pain: a Randomized Controlled Trial. Arch Phys Med Rehabil.

[76] Sale P, Mazzoleni S, Lombardi V, Galafate D, Massimiani MP, Posteraro F, Damiani C, Franceschini M (2014). Recovery of hand function with robot-assisted therapy in acute stroke patients: a randomized-controlled trial. Int J rehabilitation Res Int Z fur Rehabilitationsforschung Revue Int de recherches de readaptation.

[77] Biggar S, Yao W (2016). Design and evaluation of a Soft and Wearable Robotic Glove for Hand Rehabilitation. IEEE Trans neural Syst rehabilitation engineering: publication IEEE Eng Med Biology Soc.

[78] Orihuela-Espina F, Roldán GF, Sánchez-Villavicencio I, Palafox L, Leder R, Sucar LE, Hernández-Franco J (2016). Robot training for hand motor recovery in subacute stroke patients: a randomized controlled trial. J hand therapy: official J Am Soc Hand Therapists.

[79] Vanoglio F, Bernocchi P, Mule C, Garofali F, Mora C, Taveggia G, Scalvini S, Luisa A (2017). Feasibility and efficacy of a robotic device for hand rehabilitation in hemiplegic stroke patients: a randomized pilot controlled study. Clin Rehabil.

[80] Nam C, Rong W, Li W, Xie Y, Hu X, Zheng Y (2017). The Effects of Upper-Limb Training assisted with an Electromyography-Driven Neuromuscular Electrical Stimulation Robotic Hand on Chronic Stroke. Front Neurol.

[81] Aprile I, Germanotta M, Cruciani A, Pecchioli C, Loreti S, Papadopoulou D, Montesano A, Galeri S, Diverio M, Falsini C (2021). Poststroke shoulder pain in subacute patients and its correlation with upper limb recovery after robotic or conventional treatment: a secondary analysis of a multicenter randomized controlled trial. Int J stroke: official J Int Stroke Soc.

[82] Shin JH, Bog Park S, Ho Jang S (2015). Effects of game-based virtual reality on health-related quality of life in chronic stroke patients: a randomized, controlled study. Comput Biol Med.

[83] Pulman J, Buckley E (2013). Assessing the efficacy of different upper limb hemiparesis interventions on improving health-related quality of life in stroke patients: a systematic review. Top Stroke Rehabil.

[84] Fernández-Vázquez D, Cano-de-la-Cuerda R, Navarro-López V. Haptic Glove Systems in Combination with Semi-Immersive Virtual Reality for Upper Extremity Motor Rehabilitation after Stroke: A Systematic Review and Meta-Analysis. Int J Environ Res Public Health 2022, 19(16). 10.3390/ijerph191610378 PMC9408073 36012019

[85] Wonsick M, Padir TJAS. A systematic review of virtual reality interfaces for controlling and interacting with robots. 2020, 10(24):9051.

[86] Guillén-Climent S, Garzo A, Muñoz-Alcaraz MN, Casado-Adam P, Arcas-Ruiz-Ruano J, Mejías-Ruiz M. Mayordomo-Riera FJJJon, rehabilitation: A usability study in patients with stroke using MERLIN, a robotic system based on serious games for upper limb rehabilitation in the home setting. 2021, 18(1):1–16. 10.1186/s12984-021-00837-z PMC7901008 33622344

[87] Zanatta F, Giardini A, Pierobon A, D’Addario M, Steca P (2022). A systematic review on the usability of robotic and virtual reality devices in neuromotor rehabilitation: patients’ and healthcare professionals’ perspective. BMC Health Serv Res.

[88] Babaiasl M, Mahdioun SH, Jaryani P, Yazdani MJD, Technology RA. A review of technological and clinical aspects of robot-aided rehabilitation of upper-extremity after stroke. 2016, 11(4):263–80. 10.3109/17483107.2014.1002539 25600057

[89] Bessler J, Prange-Lasonder GB, Schulte RV, Schaake L, Prinsen EC, Buurke JH (2020). Occurrence and type of adverse events during the Use of Stationary Gait Robots-A systematic literature review. Front Rob AI.

[90] Housman SJ, Scott KM, Reinkensmeyer DJ (2009). A randomized controlled trial of gravity-supported, computer-enhanced arm exercise for individuals with severe hemiparesis. Neurorehabilit Neural Repair.

[91] Milot MH, Spencer SJ, Chan V, Allington JP, Klein J, Chou C, Bobrow JE, Cramer SC, Reinkensmeyer DJ (2013). A crossover pilot study evaluating the functional outcomes of two different types of robotic movement training in chronic stroke survivors using the arm exoskeleton BONES. J Neuroeng Rehabil.

[92] Page SJ, Hill V, White S (2013). Portable upper extremity robotics is as efficacious as upper extremity rehabilitative therapy: a randomized controlled pilot trial. Clin Rehabil.

[93] Cimolin V, Germiniasi C, Galli M, Condoluci C, Beretta E, Piccinini LJJoD, Disabilities P. Robot-Assisted upper limb training for hemiplegic children with cerebral palsy. 2019, 31(1):89–101.

[94] Krebs HI, Saitoh E, Hogan N (2015). Robotic therapy and the Paradox of the diminishing number of degrees of Freedom. Phys Med Rehabil Clin N Am.

[95] Bessler J, Prange-Lasonder GB, Schaake L, Saenz JF, Bidard C, Fassi I, Valori M, Lassen AB, Buurke JH (2021). Safety Assessment of Rehabilitation Robots: a review identifying Safety Skills and current knowledge gaps. Front Rob AI.

[96] Abu-Dakka FJ, Valera A, Escalera JA, Abderrahim M, Page A, Mata V. Passive Exercise Adaptation for Ankle Rehabilitation Based on Learning Control Framework. Sens (Basel) 2020, 20(21). 10.3390/s20216215 PMC7662251 33142669

[97] Martí Carrillo F, Butchart J, Knight S, Scheinberg A, Wise L, Sterling L. McCarthy CJAToH-RI: Adapting a general-purpose social robot for paediatric rehabilitation through in situ design. 2018, 7(1):1–30.

[98] Chisholm KJ, Klumper K, Mullins A, Ahmadi M (2014). A task oriented haptic gait rehabilitation robot. Mechatronics.

[99] Nielsen J (2012). Usability 101: introduction to usability.

[100] Monardo G, Pavese C, Giorgi I, Godi M, Colombo R (2021). Evaluation of patient motivation and satisfaction during technology-assisted Rehabilitation: an Experiential Review. Games for health journal.

[101] Tousignant M, Boissy P, Moffet H, Corriveau H, Cabana F, Marquis F, Simard JJT. e-Health: Patients’ satisfaction of healthcare services and perception with in-home telerehabilitation and physiotherapists’ satisfaction toward technology for post-knee arthroplasty: an embedded study in a randomized trial. 2011, 17(5):376–82. 10.1089/tmj.2010.0198 21492030

[102] Andrade RM, Sapienza S, Bonato P. Development of a “transparent operation mode” for a lower-limb exoskeleton designed for children with cerebral palsy. In: *2019 IEEE 16th international conference on rehabilitation robotics (ICORR): 2019*: IEEE; 2019: 512–517. 10.1109/ICORR.2019.8779432 31374681

[103] Wang J, Li J, Zhang Y, Wang S. Design of an exoskeleton for index finger rehabilitation. In: *2009 Annual International Conference of the IEEE Engineering in Medicine and Biology Society: 2009*: IEEE; 2009: 5957–5960. 10.1109/IEMBS.2009.5334779 19965067

[104] Sampson M, Shau YW, King MJ (2012). Bilateral upper limb trainer with virtual reality for post-stroke rehabilitation: case series report. Disabil rehabilitation Assist Technol.

[105] Zhang K, Chen X, Liu F, Tang H, Wang J, Wen W. System Framework of Robotics in Upper Limb Rehabilitation on Poststroke Motor Recovery. *Behavioural neurology* 2018, 2018:6737056. 10.1155/2018/6737056 PMC6311736 30651892

[106] He Y, Eguren D, Luu TP, Contreras-Vidal JL (2017). Risk management and regulations for lower limb medical exoskeletons: a review. Med devices (Auckland NZ).

[107] Akiyama Y, Yamada Y, Ito K, Oda S, Okamoto S, Hara S. Test method for contact safety assessment of a wearable robot -analysis of load caused by a misalignment of the knee joint. In: *2012 IEEE RO-MAN: The 21st IEEE International Symposium on Robot and Human Interactive Communication: 9–13 Sept. 2012 2012*; 2012: 539–544.

[108] Melia M, Geissler B, König J, Ottersbach HJ, Umbreit M, Letzel S, Muttray A (2019). Pressure pain thresholds: subject factors and the meaning of peak pressures. Eur J Pain.

[109] Ferreira F, Chaves MEA, Oliveira VC, Van Petten A, Vimieiro CBS (2018). Effectiveness of robot therapy on body function and structure in people with limited upper limb function: a systematic review and meta-analysis. PLoS ONE.

[110] Lohse KR, Lang CE, Boyd LA (2014). Is more better? Using metadata to explore dose-response relationships in stroke rehabilitation. Stroke.

[111] Hayward KS, Brauer SG (2015). Dose of arm activity training during acute and subacute rehabilitation post stroke: a systematic review of the literature. Clin Rehabil.

[112] Kwakkel, GJp. Intensity of practice after stroke: More is better. 2009, 7:24.

[113] Wilson RD, Chae J (2015). Hemiplegic Shoulder Pain. Phys Med Rehabil Clin North Am.

[114] Tran DA, Pajaro-Blazquez M, Daneault JF, Gallegos JG, Pons J, Fregni F, Bonato P, Zafonte R (2016). Combining Dopaminergic Facilitation with Robot-Assisted Upper Limb Therapy in Stroke Survivors: a focused review. Am J Phys Med Rehabil.

[115] Kafri M, Myslinski MJ, Gade VK, Deutsch JE (2014). Energy expenditure and exercise intensity of interactive video gaming in individuals poststroke. Neurorehabil Neural Repair.

[116] Cuesta-Gómez A, Sánchez-Herrera-Baeza P, Oña-Simbaña ED, Martínez-Medina A, Ortiz-Comino C, Balaguer-Bernaldo-de-Quirós C, Jardón-Huete A. Cano-de-la-Cuerda RJJon, rehabilitation: Effects of virtual reality associated with serious games for upper limb rehabilitation in patients with multiple sclerosis: Randomized controlled trial. 2020, 17(1):1–10. 10.1186/s12984-020-00718-x PMC7359450 32660604

[117] Laut J, Porfiri M, Raghavan P (2016). The Present and Future of Robotic Technology in Rehabilitation. Curr Phys Med Rehabilitation Rep.

[118] Francisco GE, Yozbatiran N, Berliner J, OʼMalley MK, Pehlivan AU, Kadivar Z, Fitle K, Boake C (2017). Robot-Assisted training of arm and Hand Movement shows functional improvements for incomplete cervical spinal cord Injury. Am J Phys Med Rehabil.

[119] Tousignant M, Boissy P, Moffet H, Corriveau H, Cabana F, Marquis F, Simard J (2011). Patients’ satisfaction of healthcare services and perception with in-home telerehabilitation and physiotherapists’ satisfaction toward technology for post-knee arthroplasty: an embedded study in a randomized trial. Telemedicine J e-health: official J Am Telemedicine Association.

[120] Shirley ED, Sanders JO (2013). Patient satisfaction: implications and predictors of success. J bone joint Surg Am volume.

[121] Monardo G, Pavese C, Giorgi I, Godi M, Colombo RJGfhj. Evaluation of patient motivation and satisfaction during technology-assisted rehabilitation: an experiential review. 2021, 10(1):13–27. 10.1089/g4h.2020.0024 32614618

[122] Kwon SH, Lee BS, Lee HJ, Kim EJ, Lee JA, Yang SP, Kim TY, Pak HR, Kim HK, Kim HYJAoRM. Energy efficiency and patient satisfaction of gait with knee-ankle-foot orthosis and robot (ReWalk)-assisted gait in patients with spinal cord injury. 2020, 44(2):131–41. 10.5535/arm.2020.44.2.131 PMC7214138 32392652

[123] Kim DJ, Hazlett-Knudsen R, Culver-Godfrey H, Rucks G, Cunningham T, Portee D, Bricout J, Wang Z, Behal A (2012). How autonomy impacts performance and satisfaction: results from a study with spinal cord injured subjects using an Assistive Robot. IEEE Trans Syst Man Cybernetics - Part A: Syst Hum.

[124] Ramos Muñoz EJ, Swanson VA, Johnson C, Anderson RK, Rabinowitz AR, Zondervan DK, Collier GH, Reinkensmeyer DJ (2022). Using large-scale Sensor Data to test factors predictive of perseverance in Home Movement Rehabilitation: optimal challenge and steady Engagement. Front Neurol.

[125] Akbari A, Haghverd F, Behbahani S (2021). Robotic home-based Rehabilitation Systems Design: from a literature review to a conceptual Framework for Community-Based remote therapy during COVID-19 pandemic. Front Rob AI.

[126] Kutner NG, Zhang R, Butler AJ, Wolf SL, Alberts JL (2010). Quality-of-life change Associated with robotic-assisted therapy to Improve Hand Motor function in patients with Subacute Stroke: a Randomized Clinical Trial. Phys Ther.

[127] Mohammed SA, Shebl AM (2014). Quality of life among egyptian patients with Upper and Lower Limb Amputation: sex differences. Adv Med.

[128] Rasouli S, Gupta G, Nilsen E, Dautenhahn K (2022). Potential applications of Social Robots in Robot-Assisted interventions for social anxiety. Int J Social Robot.

[129] Kawatsuma S, Mimura R, Asama H (2017). Unitization for portability of emergency response surveillance robot system: experiences and lessons learned from the deployment of the JAEA-3 emergency response robot at the Fukushima Daiichi Nuclear Power plants. ROBOMECH J.

[130] Treviño LR, Roberge P, Auer ME, Morales A, Torres-Reveron A (2021). Predictors of functional outcome in a cohort of hispanic patients using Exoskeleton Rehabilitation for Cerebrovascular Accidents and Traumatic Brain Injury. Front Neurorobotics.

