# Supplementary material for: Overview of the role of robots in upper limb disabilities rehabilitation: a scoping review
Source: Arch Public Health. 2023 May 8;81:84. doi: 10.1186/s13690-023-01100-8 (PMC10169358; doi:10.1186/s13690-023-01100-8)
Supplement: Supplementary file 1 — Supplementary Material 1 Aims and scope statement [file 13690_2023_1100_MOESM1_ESM.docx]

**What is known:** Impaired upper limb (UL) function restricts performance of activities of daily living, limits social participation (1), specifically decreases the independence of affected individuals and reduces patient’s quality of life (2, 3). In upper limb disabilities, patients develop musculoskeletal problems such as paresis, pain, loss of sensation, and spasticity in different parts of the upper limb and so, these problems can manifold consequences in the daily lives of those impacted. These include a decreased capacity to carry out primary self-care tasks and to accomplish life-roles, which can affect emotional, mental, and psychological wellbeing (4). Patients with upper limb disabilities need rehabilitation to improve their musculoskeletal status (5). New therapeutic methods have been presented to rehabilitate and improve upper limb function, and such methods are robotic rehabilitation (1).

**What does the study adds:** Our scoping review indicates that upper limb rehabilitation robots are able to improve musculoskeletal functions (Musculoskeletal strength, sensation, perception, vibration, muscle coordination, less spasticity, flexibility, and range of motion); avoid serious side effects or adverse effects on patient, provide safe and reliable treatment, reduce pain, increase the patient's independence in performing rehabilitation exercises, reduce the duration of rehabilitation exercises, increase adherence to rehabilitation exercises and treatment processes, and quality of life and rehabilitation processes. The use of rehabilitation robots is increasing strongly, especially in developed countries, and it seems that this new rehabilitation technology has had significant effects in helping to improve upper limb disabilities. Also, rehabilitation robots could provide a platform for motivating people with upper limb disabilities to carry out more rehabilitation exercises in the absence of a therapist, which could maximize recovery.

**What are implications for clinical practice, public health and / or research:** The different aspects of upper limb rehabilitation robots identified in this study can be used to design and develop rehabilitation robots for other disabilities. Moreover, due to the identification of various application and benefits of rehabilitation robots, this study can increase the motivation of therapists, policymakers, and health planners to use and use more rehabilitation robots in medical centers and hospitals. In addition, this study can provide background and basic knowledge related to rehabilitation robots for future research.

**References**

1. Lee SH, Park G, Cho DY, Kim HY, Lee J-Y, Kim S, et al. Comparisons between end-effector and exoskeleton rehabilitation robots regarding upper extremity function among chronic stroke patients with moderate-to-severe upper limb impairment. Scientific Reports. 2020;10(1):1806.

2. Vélez-Guerrero MA, Callejas-Cuervo M, Mazzoleni S. Artificial Intelligence-Based Wearable Robotic Exoskeletons for Upper Limb Rehabilitation: A Review. Sensors (Basel). 2021;21(6):2146.

3. Moulaei K, Sheikhtaheri A, Nezhad MS, Haghdoost A, Gheysari M, Bahaadinbeigy K. Telerehabilitation for upper limb disabilities: a scoping review on functions, outcomes, and evaluation methods. Archives of Public Health. 2022;80(1):196.

4. Poltawski L, Allison R, Briscoe S, Freeman J, Kilbride C, Neal D, et al. Assessing the impact of upper limb disability following stroke: a qualitative enquiry using internet-based personal accounts of stroke survivors. 2016;38(10):945-51.

5. Maciejasz P, Eschweiler J, Gerlach-Hahn K, Jansen-Troy A, Leonhardt S. A survey on robotic devices for upper limb rehabilitation. Journal of NeuroEngineering and Rehabilitation. 2014;11(1):3.
