# Supplementary material for: Overview of the role of robots in upper limb disabilities rehabilitation: a scoping review
Source: Arch Public Health. 2023 May 8;81:84. doi: 10.1186/s13690-023-01100-8 (PMC10169358; doi:10.1186/s13690-023-01100-8)
Supplement: Supplementary file 2 — Supplementary Material 2: Appendix A [file 13690_2023_1100_MOESM2_ESM.docx]

**Appendix A**: Quality Assessment of Included Studies Using the MMAT Criteria

| **Ref** | **Quantitative descriptive** | | | | | **Quantitative randomized controlled trials** | | | | |
| --- | --- | --- | --- | --- | --- | --- | --- | --- | --- | --- |
|  | **Is the sampling strategy relevant to address the research question?** | **Is the sample representative of the target population?** | **Are the measurements appropriate?** | **Is the risk of nonresponse bias low?** | **Is the statistical analysis appropriate to answer the research question?** | **Is randomization appropriately performed?** | **Are the groups comparable at baseline?** | **Are there complete outcome data?** | **Are outcome assessors blinded to the intervention provided?** | **Did the participants adhere to the assigned intervention?** |
| Hwang [26] |  |  |  |  |  | Y | Y | Y | Y | Y |
| Carpinella [27] | Y | Y | Y | CT | Y |  |  |  |  |  |
| Hu [28] | Y | Y | Y | Y | Y |  |  |  |  |  |
| Squeri [29] | Y | Y | Y | Y | Y |  |  |  |  |  |
| Sale[30] |  |  |  |  |  | Y | CT | Y | CT | Y |
| Sale[31] | Y | Y | Y | CT | Y |  |  |  |  |  |
| Klamroth-Marganska [32] |  |  |  |  |  | Y | Y | Y | Y | Y |
| Hsieh [33] | Y | Y | Y | CT | Y |  |  |  |  |  |
| Pennati [34] |  |  |  |  |  | Y | Y | Y | Y | Y |
| McCabe [35] |  |  |  |  |  | Y | CT | Y | Y | Y |
| Chen[36] | CT | Y | Y | Y | Y |  |  |  |  |  |
| Vanmulken[37] | Y | Y | Y | N | Y |  |  |  |  |  |
| Gilliaux [38] |  |  |  |  |  | CT | Y | Y | Y | Y |
| Taveggia [39] |  |  |  |  |  | Y | Y | Y | CT | Y |
| Biggar[40] | Y | Y | Y | CT | Y |  |  |  |  |  |
| Orihuela-Espina [41] |  |  |  |  |  | Y | Y | Y | Y | Y |
| Song [42] | Y | Y | Y | Y | Y |  |  |  |  |  |
| Vanoglio [43] |  |  |  |  |  | Y | Y | Y | Y | Y |
| Trujillo [44] | Y | Y | Y | Y | Y |  |  |  |  |  |
| Saita [45] | Y | Y | Y | Y | Y |  |  |  |  |  |
| Nam[46] |  |  |  |  |  | Y | Y | CT | Y | Y |
| McKenzie [47] | Y | Y | Y | Y | Y |  |  |  |  |  |
| Kim [48] |  |  |  |  |  | Y | Y | Y | Y | Y |
| Bishop [49] | Y | Y | Y | CT | Y |  |  |  |  |  |
| Housley [50] | Y | Y | Y | CT | Y |  |  |  |  |  |
| Hsieh [51] |  |  |  |  |  | Y | Y | Y | Y | Y |
| Gandolfi [52] |  |  |  |  |  | CT | Y | Y | CT | Y |
| Lee [53] | CT | Y | Y | CT | Y |  |  |  |  |  |
| Germanotta [54] | Y | Y | Y | Y | Y |  |  |  |  |  |
| Kim [55] | Y | Y | Y | Y | Y |  |  |  |  |  |
| Villafañe [56] |  |  |  |  |  | Y | Y | Y | Y | Y |
| Palermo [57] | Y | Y | Y | Y | Y |  |  |  |  |  |
| Iwamoto [58] | Y | Y | Y | Y | Y |  |  |  |  |  |
| Kim [59] |  |  |  |  |  | Y | Y | Y | Y | Y |
| Dehem [60] |  |  |  |  |  | Y | Y | CT | Y | Y |
| Hung [61] |  |  |  |  |  | Y | Y | Y | Y | Y |
| Conroy [62] |  |  |  |  |  | Y | Y | Y | CT | Y |
| Bonanno [63] | Y | Y | Y | Y | Y |  |  |  |  |  |
| Leem [64] | Y | Y | Y | CT | Y |  |  |  |  |  |
| Kim [65] | Y | Y | Y | CT | Y |  |  |  |  |  |
| Tartamella [66] | Y | Y | Y | CT | Y |  |  |  |  |  |
| Solaro [67] |  |  |  |  |  | CT | Y | Y | Y | Y |
| Picelli [68] |  |  |  |  |  | Y | Y | Y | Y | Y |
| Kuo [69] | Y | Y | Y | Y | Y |  |  |  |  |  |
| Aprile [70] |  |  |  |  |  | Y | Y | Y | CT | Y |
| Aprile [71] | Y | Y | Y | CT | Y |  |  |  |  |  |
| Bouteraa [72] | CT | Y | Y | N | Y |  |  |  |  |  |
| Kim [73] |  |  |  |  |  | Y | Y | Y | Y | Y |
| Bui [74] | Y | Y | Y | CT | Y |  |  |  |  |  |
| Flynn [75] | CT | Y | Y | CT | Y |  |  |  |  |  |
| Terranova [76] |  |  |  |  |  | Y | Y | Y | CT | Y |
| Shi [77] | Y | Y | Y | Y | Y |  |  |  |  |  |
| Chen [78] |  |  |  |  |  | Y | Y | CT | Y | Y |
| Qu [79] |  |  |  |  |  | Y | Y | Y | Y | Y |
| Abd [80] |  |  |  |  |  | Y | CT | Y | Y | Y |

**^*^Note**: Abbreviations: Y = yes; N = no; CT = can’t tell; RQs: research question.
