# Supplementary material for: Overview of the role of robots in upper limb disabilities rehabilitation: a scoping review
Source: Arch Public Health. 2023 May 8;81:84. doi: 10.1186/s13690-023-01100-8 (PMC10169358; doi:10.1186/s13690-023-01100-8)
Supplement: Supplementary file 3 — Supplementary Material 3: Appendix B [file 13690_2023_1100_MOESM3_ESM.docx]

**Appendix B**: Overview of the outcomes and evaluation methods of rehabilitation robots presented in the studies

| **Ref** | **Number of participants in the study** | **Sex** | | **Age or mean of age (SD)** | **Rehabilitated part of the upper limb using robot** | **Duration and frequency of treatment** | **Methods of performing rehabilitation exercises** | **Evaluation Methods/tools** | **Evaluation outcomes** | **Study outcomes** |
| --- | --- | --- | --- | --- | --- | --- | --- | --- | --- | --- |
|  |  | **Male** | **Female** |  |  |  |  |  |  |  |
| Hwang [26] | Fifteen patients with stroke ((the FTI (full-term intervention) group, 9 patients) and (the HTI (half-term intervention) group, 6 patients)) | ✓  (n=9) | ✓  (n=6) | ≥18 years | Fingers | -Four weeks (20 sessions) for FTI group  -Two weeks (10 sessions) for HTI | - FTI group received active robot-assisted intervention for performing grasping and releasing exercises  - HTI group received passive range of motion training for wrist and hand movement and proximal arm movement function | Fugl-Meyer Upper Extremity score (FMA-UE) and pegboard test | - Improved outcomes for the Jebsen Taylor test, the wrist and hand sub-portion of the Fugl-Meyer arm motor scale, active motion of the 2nd meta carpophalangeal joint, grasping, and pinching powerfulness (P < 0.05 for all) both the FTI and HTI groups  -A larger degree of advance for the FTI compared to the HTI group (P < 0.05); for example, in Jebsen Taylor test (65.9 ± 36.5 vs. 46.4 ± 37.4) and wrist and hand sub-portion of the Fugl-Meyer arm motor scale (4.3 ± 1.9 vs. 3.4 ± 2.5) after eight weeks | -Improvement in hand function in subacute to chronic stroke patients  -Provides individual finger synchronization in a dose-dependent by using a novel robot |
| Carpinella [27] | Twenty-two MS patients | ✓  (n=10) | ✓  (n=12) | 50.8(9.6) | Arm | Eight sessions with epochs of 20 movements each (a total of 160 movements and a duration of 30–45 min) | - Carry the arm by moving the handle of a planar robotic manipulator toward circular targets on the screen | Nine Hole Peg test (9 HPT), Action Research Arm Test (ARAT), and Tremor Severity Scale (TSS) | - A significantly larger improvement in movements involving grasp (improvement in Grasp ARAT sub-score: RMT 77.4%, RT 29.5%, p=0.035) but not precision grip in RMT protocol compared to RT  - Obtain a percent advance of Grasp sub-score significantly in RMT group higher than that attained by RT group  - Significantly increase of the execution frequency of ARAT tasks after the treatment in both groups  - Similar advance in both groups for execution frequency of Grip [RT: 18.1% (17.8%); Pinch items [RT: 17.2% (20.0%); RMT: 17.8% (34.0%)], and RMT: 21.6% (19.8%)]  - Similarity of The percentage change obtained after the training in both groups [RT: 14.1% (16.3%); RMT: 12.1% (19.3%)]  - An improvement greater than 20% in three subjects in the RT group and five subjects in the RMT group | - Increase the rate of motor learning of patients with the help of robots  - Significantly reduced arm vibration and improved arm kinematics and functional ability by Robot-therapy  -Improve upper limb kinematics and functional ability in subjects with MS |
| Hu [28] | Ten patients with chronic stroke | ✓  (n=7) | ✓  (n=3) | 18 to 78 years | Fingesr and wrist | Twenty session training (3-5 times/week) | -Grasp and release a sponge along a table using the robot to perform arm and wrist and fingers rehabilitation exercises | Fugl-Meyer, Action Research Arm Test (ARAT), MAS on the flexors related to the elbow, wrist, and fingers, and Wolf Motor Function Test (WMFT) | - Significant motor improvements in the Fugl-Meyer hand/wrist and shoulder/elbow scores (p<0.05), and also in the Action Research Arm Test and Wolf Motor Function Test (p<0.05)  - Significant reduction in spasticity of the fingers according to Modified Ashworth Score (p<0.05)  - Improve the muscle coordination between the antagonist muscle pair (flexor digitorum (FD) and the extensor digitorum (ED)), associated with a significant reduction in the ED EMG level (p<0.05) and a significant decrease of ED and FD co-contraction during the training (p<0.05)  - Reduce significantly of the excessive muscle activities in the biceps brachii after the training (p<0.05) | - Improve the muscle coordination by EMG-driven robot  - Monitoring the variations in the muscular coordination patterns by EMG parameters  - Significant reduction in the EMG levels in the flexor digitorum and the biceps brachii |
| Squeri [29] | Nine stroke patients | ✓  (n=2) | ✓  (n=7) | 29 to 72 years | Wrist and forearm | Five weeks (ten 1-hour sessions) | -Use of a haptic three DoFs (degrees of freedom) robot to quantify motor disabilities and assist wrist and forearm articular movements | Range of motion (RoM), Fugl-Meyer assessment (FMA) and Wolf Motor Function Test (WOLF) | - A significant improvement (average of 9.33±1.89 points) in FMA score  - Reduction of the upper extremity motor impairment  - A remarkable improvement inactive RoM  - Improvement of 8.31±2.77 in WOLF (highlighting an increase in functional capability for the whole arm) | - Recover and improve motor functions for distal and proximal limb sectors  - A positive trend and substantial differences over the whole protocol and follow-up too |
| Sale[30] | Twenty acute stroke patients in control and experimental groups | ✓  (n=14) | ✓  (n=6) | Patients younger than 18 years and older than 80 years of  age | Hand | Twenty sessions (4/5 days a week for 4/5 weeks) | -Experimental group: Performing hand flexion and extension rehabilitation exercises with the robot  -Control group: Performing hand flexion and extension rehabilitation exercises with the help of a trained physiotherapist | Fugl-Meyer Scale (FM), Medical Research Council Scale for Muscle Strength (hand flexor and extensor muscles) (MRC), Motricity Index (MI) and modified Ashworth Scale for wrist and hand muscles (MAS) | - Show a significant improvement by the Friedman test for the FM [experimental group (EG): P=0.0039, control group (CG): P<0.0001], Box and Block Test (EG: P=0.0185, CG: P=0.0086), MI (EG: P<0.0001, CG: P=0.0303) and MRC (EG: P<0.0001, CG: P=0.001) scales  - A statistically significant improvement in EG for the FM (P = 0.0039), the BB (P = 0.0185), MI (P < 0.0001), and MRC (P < 0.0001)  - A statistically significant increase in the MAS score in EG was found (P = 0.0025)  - Statistically significant improvements in CG for the FM (P < 0.0001), BB (P = 0.0086), MI (CG: P = 0.0303), and MRC (CG: P = 0.001) | -Hand functions recovery in individuals with acute stroke  - Safe and reliable treatment through rehabilitation robots |
| Sale[31] | Thirteen patients | ✓  (n=9) | ✓  (n=4) | 53.2(13.5) | Upper limb | Six-month (20 treatment sessions lasting 45 min each, 5 days a week, for a total period of 4 weeks, repeated twice, for a total of 40 sessions) | -Performing rehabilitation exercises such as forward thrust, forward reach 2D and 3D, horizontal reach, horizontal abduction, and functional with the help of a robot | Fugl-Meyer test, the Ashworth Scale test, the Frenchay Arm test, and the Box and Block test according to the following schedule: immediately before (T1, T3) and after each treatment (T2, T4), and 6 months after T4 (T5) | - Statistically significant improvements in Fugl-Meyer test between T1 and T2 and between T1 and T4  - Increase the score in the Ashworth Scale test for Shoulder between T1 and T3 and between T1 and T5  - A statistically significant decrease between T1 and T2 and between T1 and T4, in the Box and Block test between T1 and T4, and also between T1 and T5 | - Increase the upper limb motor recovery in chronic stroke patients |
| Klamroth-Marganska [32] | Seventy-three patients (35 received conventional therapy and 38 received robotic therapy) | ✓  (n=46) | ✓  (n=27) | 18–80 years | Arm, elbow and shoulder | More than 6 months (for at least 45 min three times a week for 8 weeks (total 24 sessions)) | -Robotic therapy: Performing rehabilitation exercises of arm and shoulder abduction, adduction, anteversion and retroversion and elbow bending and extension with the robot  -Conventional therapy group: Performing rehabilitation exercises in a conventional way with the help of a therapist | Fugl-Meyer assessment (FMA-UE) and Wolf Motor Function Test | - Significantly changes in FMA-UE scores in patients assigned to robotic therapy than conventional therapy  - The mean change over the whole course FOR patients assigned to robotic therapy= 3.25 points (SD 1.68)  -The mean change over the whole course for patients assigned to conventional therapy= 2.47 points (SD1.67) | -No serious side-effects in robotic therapy for patient  -Improvement in motor function |
| Hsieh [33] | Fifty-five with stroke | ✓  (n=35) | ✓  (n=20) | 23-77 years | Wrist and forearm | Patients with stroke received RT for 90 to 105min/d, 5d/wk, for 4 weeks. | - Receiving robot-assisted therapy (RT)by patients for enabling forearm pronation-supination and wrist flexion-extension movements in 3 computer-controlled ways: passive-passive, active-active, and active-passive | Fugl-Meyer Assessment (FMA), Motor Activity Log (MAL) and Box and Block Test (BBT) score | - BBT score (odds ratio [OR]=1.06; P=.04) as a significant predictor of clinically important changes in the FMA  - Being a woman (OR=3.9; P=.05) and BBT score (OR=1.07; P=.02) as 2 significant predictors of clinically significant changes in the MAL amount of use subscale  - The BBT score as a significant predictor of an increased probability of achieving clinically important changes in the MAL quality of movement subscale (OR=1.07; P=.02)  - Lowing the R2 values for the 3 logistic regression models low (.114−.272) | - Significantly improvement in hands motor and functional outcomes after RT |
| Pennati [34] | Fifteen chronic post-strokes in two experimental groups (A and B gropus) | ✓  (n=9) | ✓  (n=6) | 18-85 years | Upper limb | Ten sessions lasting 60 minutes each, 2 or 3 days a week | -Group A: Performing two movements: one movement to reach the wall (shoulder flexion, elbow extension, wrist extension) and return to the mouth (elbow and wrist flexion with shoulder return from flexion), and reaching the movement towards the visual target with the help of Robot in assisted and unassisted mode  - Group B: following the botulinum toxin type A neurolysis of upper extremity spastic muscles, within few days, by the same robotic training | Fugl Meyer Upper Limb Assessment Scale (FMA) and Box & Block Test (B&B), disability with Functional Indipendence Measure (FIM), spasticity with Modified Ashworth Scale (MAS), and the Quality of Life (Euro-Qol) and Dynamic Surface Electromyography (sEMG) | - Improvement in FMA in both groups (Group A 8.25 and Group B 5.29)  - Higher improvement in B&B in the group A (2.62 versus 0,14 in Group B)  - Improve the MAS more in the Group B (-0,86 versus -0,14 in Group A)  - A reduction of co-contractions and an increase of agonist muscle recruitment during the reaching movement and the robotic exercises in both group | -Improvement in motor function and in muscular activation pattern  - Effectiveness of robotic training in reduction chronic post-stroke spasticity of upper limb |
| McCabe [35] | Thirty-five patients with stroke | ✓  (n=23) | ✓  (n=12) | 50–81 years | Upper limb | 5d/wk for 5h/d (60 sessions) | -Carrying out the rehabilitation components of reaching, grasp preparation, and grasp release with the help of a robot in order to increase the coordination of movement ranges and restore functional tasks | Arm Motor Ability Test (AMAT), Fugl Meyer coordination scale (FM) | - No significant difference found in treatment response across groups (AMAT: P≥.584; FM coordination: P≥.590)  - Clinically and statistically significant improvement in response to treatment (AMAT and FM coordination: P≤.009) in all 3 treatment groups | - Coordination and functional task performance in response to robotics plus ML, FES plus ML, and ML alone in an intensive and long-duration intervention |
| Chen[36] | A healthy subject | Not mentioned | Not mentioned | 18-70 years | Wrist and arm | Not mentioned | - Performing wrist and arm rehabilitation exercises with a robot | Performance of the controller and the feasibility of the cable-driven wrist robotic rehabilitator (CDWRR) | - Significant improvements in joint motion tracking accuracy with the assistance of the CDWRR, comparing the angle errors in 17(a) and 17(b)  - Angle errors of wrist motion are less than 5◦ with the assistance from the DEER, and the angle errors are lesser than 5◦ and no more than 10◦ without the assistance | -Improve the wrist motion accuracy |
| Vanmulken[37] | Five C-SCI patients | ✓  (n=4) | ✓  (n=1) | 18-70 years | Arm and hand | Six weeks, 3 days per week, 60 min per day | -Providing training/or exercises such as 'eating with fork and knife', 'taking money out of a purse' and 'moving a cup' to the patient with the help of a robot | - Usefulness, Satisfaction and Ease-of-use questionnaire (USE), Motivational Inventory (IMI), credibility and expectancy questionnaire (CEQ), Van Lieshout test for arm–hand function (AHF), Spinal Cord Independence | -Mean 67% and 60% for IMI and CEQ results  -The mean Usefulness, Satisfaction and Ease-of-use questionnaire results, rated by the therapists, amounted to 71.0% (s.d.: 13.4%) for the item ‘ease of use’, 93.9% (s.d.: 7.5%) for the item ‘easy to learn’, 59.2% (s.d.: 25.5%) for ‘satisfaction’ and 47.0% (s.d.: 18.4%) for ‘usefulness  - Mean 65.1% (s.d .: 5.0%) for total score of the Usefulness, Satisfaction and Ease-of-use  - No large improvements at activity level  - -High score on ‘self care’ and ‘respiration and sphincter management’ for Spinal Cord Independence Measure in all participants  -Low score on ‘mobility’ for Spinal Cord Independence Measure in all participants  - Improve the muscle strength on all major arm muscles (improvement ranging between 8% and 22%)  - Improve the elbow flexion (23%), shoulder abduction (13%) and wrist extension (17%). | -Increase participants’ motivation to train with the Haptic Master (HM)  -Feasibility of C-SCI with robotic systems to train persons |
| Gilliaux [38] | Sixteen children with CP in robotic and control group | Not mentioned. | Not mentioned. | 10.8 (4.6) | Upper limb | Five sessions of therapy per week over the course of 8 weeks (40 sessions in all) for both groups | -Robotic group: Drawing a square of 6 cm side and a circle of 4 cm radius with the robot  -Control group: receiving eight sessions of conventional therapy | Box and Block test, Quality of Upper Extremity Skills Test (QUEST), Abilhand-Kids | - Significantly improvement in the smoothness of movement (P < .01) and manual dexterity assessed by the Box and Block test (P = .04) more in the robotic group than in the control group  - Dont change in the Control group (0.46 [0.05] to 0.46 [0.06]) for the speed metric index  - Don’t change in Free Amplitude, Square, and Circle, the kinematics indices after intervention (P > .05)  - Improve the BB score from 13.0 (7.3) to 16.6 (9.9) blocks/min in the Robotic group  - Improvement in the capacity to perform analytical movements of the upper limb similarly in both groups (P < .05)  - Significantly increase the scores of the dissociated movement’s subscales of the QUEST for the Robotic (median increased from 37.0 to 63.3/100) and Control (median increased from 44.4 to 68.8) groups (P < .04) | - Improved upper limb kinematics and manual dexterity  - Robotic devices as a fun and intensive rehabilitation for children |
| Taveggia [39] | Fifty-four patients with hemiparesis after stroke in two experimental and control groups | ✓  (n=23) | ✓  (n=31) | 18-80 years | Upper limb | Six consecutive weeks (5 days/week and 30 minutes per session) for each of the control and experimental groups | -Experimental group: receiving a passive mobilization of the upper limb through the robotic device ARMEO Spring  -Control group: receiving a physical and rehabilitation medicine (PRM) in addition to traditional PRM | Functional Independence Measure [FIM], Motricity Index (MI), Modified Ashworth Scale (MAS), Numeric Rating Pain Scale (NRPS) | - Improvement of the outcomes after the treatment in both control and experimental groups (MI, Ashworth and NRPS with P<0.05)  - Further improvements after the follow up in experimental group (all outcomes with P<0.01) | - Significantly improvement in upper limb motor function in stroke patients  - Recovery of disability, pain and spasticity in upper limb after stroke  - Safe and reliable treatment by robotic |
| Biggar[40] | A healthy participant | ✓  (n=1) |  | Not mentioned | Hand | Not mentioned. | -Grasping and lifting a selection of small to medium-sized spheres and cubes by robot | Design’s feasibility | -Increased rotation of the MCP to complete the grasping of the marble  - Decreased activity in DIP when the user wears gloves with a range of movement from 23.44-40.84° for index finger without the device, dropping to 7.61-14.64° with the device  - Increases the activity to 25.61-41.51° for the 3D printed hand this  - A larger percentage contribution from the MCP (rising from 11.21 to 42.9% for grasping a cricket ball) and a reduction in contribution from the DIP (falling from 49.28 to 19.64%) | - Confirm the feasibility and validity of the designed system  - Successful in the treatment of stroke patients by designed robot |
| Orihuela-Espina [41] | Subacute stroke patients (n = 17) in case and controls groups | ✓  (n=11) | ✓  (n=6) | >30 years | Hand | Forty sessions ensuring at least 300 repetitions per session for five times a week | -Case group: receiving treatments, whether occupational or robotic, under the supervision of an occupational therapist (First passive activities (300 repetitions), followed by assisted or partially resisted (300 repetitions), from the fourth session onwards active movements (100 repetitions) for a total of 700 repetitions per session)  -Control groups: receiving classical occupational therapy with massage and conventional occupational exercises (A warm-up phase with strengthening exercises, and a final active training phase to improve palmar awareness, and individual ball and screw activities to control fine grips (lateral and pulpal).) | Fugl-Meyer (FMA) and the Motricity Index (MI) | - Significant improvements over time (Non-parametric Cliff’s delta-within effect sizes: dwOT-FMA = 0.5, dwOT-MI = 0.5, dwRT-FMA = 1, dwRT-MI = 1) in Both groups (OT: n = 8; RT: n = 9)  - Significant advantage for the hand training with the robot (FMA hand: WRS: W = 8, p <0.01)  - Greater improvement (size effect) in hand prehension for RT with respect to OT(MI prehension: W = 17.5, p = 0.080)  - Significant advantage for the hand training with the robot (FMA hand: WRS: W = 8, p <0.01), whilst the Motricity index suggested a | - Significant improvements in hand motor dexterity by Robotic intervention in subacute stage  - Giving patients an additional opportunity to recover by robotic intervention |
| Song [42] | Four patients with upper limb disabilities | ✓  (n=3) | ✓  (n=1) | 21-53 years | Upper limb | Four weeks | -Prescribing rehabilitation exercises through the telerehabilitation system and performing exercises with the help of a robot by patients | Reliability and efficiency | - Patient 1=Improved arm mobility compared to the first days, drive the robot arm swing more quickly with wider range than four weeks before, Replacing the active training mode with damping force to replace the single training mode  - Patient 2= Increased patient arm strength 2 more than four weeks ago, selected active training mode with the maximum damping force of 45 N for patient, gradually calm the patient’s emotions and focus more on rehabilitation exercises  - Patient 3=Gradually calm the patient’s emotions and focus more on rehabilitation exercises,move the robot arm in a small range without any auxiliary force by the right arm | - Increases the muscle strength and movement coordination of the three patients after four weeks of periodic rehabilitation training  - Improve efficiency of the rehabilitation training  - Increases the enthusiasm of patients during process of rehabilitations by game therapy |
| Vanoglio [43] | Thirty hemiplegic stroke patients (Treatment group (TG) and Control group (CG)) | ✓  (n=14) | ✓  (n=16) | > 18 years | Hand and fingers | Six weeks (30 sessions, lasting 40 min/ day, for 5 days/week.) | -Treatment group: receiving intensive hand training with hand rehabilitation robot  -Control group: receiving the conventional hand rehabilitation  The trainings for both groups were: flexion-extension of the fingers, adduction and abduction of the fingers, thumb-finger opposition movements from the 2nd to the 5th finger, and fist opening/closing. | -Hand function: Motricity Index (MI), Nine Hole Peg Test, Grip and Pinch test  -Efficacy: Motricity Index (MI), Nine Hole Peg Test, Grip and Pinch test, Quick version of the Disabilities of the Arm, Shoulder, and Hand (Quick-DASH)  -Cost analysis: based on the time required by health care providers, using the mean cost per 60 minutes of a physiotherapist per total number of rehabilitation treatments per patient and in terms of the time necessitated by physiotherapist to utilize care that the robotic system working right during the sessions | -Increase in FIM scores [Control Group: 58 (32), p = 0.001; Treatment Group: 79 (31), p = 0.0005] and MotorFIM [Control Group: 35 (24), p = 0.001; Treatment Group: 53 (25), p = 0.0005] compared to baseline in both groups  -No significant changes in Ashworth spasticity index in different areas compared to baseline in both groups after inpatient rehabilitation  - Spatiality 0.46 (0.52) in control group (p = 1) and 0.57 (0.85) in treatment group (p = 1) for finger flexor  - Spatiality 0.15 (0.37) in control group (p = 1) and 0.07 (0.27, respectively) in treatment group (p = 0.5) for opponents of the thumb  - Spatiality 0.46 (0.66) in control goup (p = 0.31) and 0.71 (0.73) in treatment group (p = 0.75) for wrist flexors  -Feasibility outcome: The mean VAS score for the first three days 5.13 (1.6) vs. 1.16 (0.26) for the last 27 days  -decreases the time commitment of the physiotherapist from 24 (8.5) min in the first 3 days to 11 (1.1) min in the last 27 days  -Efficacy outcome: similarity of Motricity Index, Nine Hole Peg Test, Grip and Pinch test  -Efficacy outcome: significantly improvement in tricity Index, Nine Hole Peg Test, Grip and Pinch test  -Normalization of strength for BMI of the paretic upper limb after inpatient rehabilitation only in Treatment Group  -Significant decrease in QuickDASH score in group therapy  -Calculated device cost for a 30-day treatment period: € 89.60 per patient  -The cost of the physiotherapist for the 30 days of treatment: 40 min x €0.40/min x 30 days = €480/ patient | -Feasibility and Efficacy of Gloreha Professional in improving hand skills and strength and reducing arm disability in patients with subacute hemiplegia  -Acceptance of the system by all patients as an effective tool  -Effectiveness of the robotic device in healing the injured arm in patients with subacute stroke |
| Trujillo [44] | Ten post-stroke patients | ✓  (n=8) | ✓  (n=2) | 31-81 years | Upper limb | Four weeks (12 training sessions, each one lasting 40 minutes) | -Using the robot to perform rehabilitation exercises such as: Hand-to-Mouth Movement (HtMM), bending the shoulder on the sagittal plane up to 90 degrees, along with the full extension of the elbow, imitating the gesture of reaching an object | Fugl-Meyer Assessment (FMA) | -A significant negative correlation between PRIT0 and the ΔFMA%  - A trend to a negative correlation between DART0 and ΔFMA% (ρ=-0.54, P=0.11)  - not correlate with neither the ΔFMA (ρ=-0.02, P=0.97)  - FMAT0 was not correlated with PRIT0 (ρ=-0.19, P=0.60), DART0 (ρ=-0.012, P=0.97), nor pdBSIT0 (ρ=0.45, P=0.19); analogously, FMAT1 was not correlated with PRIT1 (ρ=-0.24, P=0.51), DART1 (ρ=-0.08, P=0.83), nor pdBSIT1 (ρ=0.42, P=0.22) | -Significant relationship between Power Ratio Index (PRI) and motor recovery  -Improving the mobility of patients with low values of Power Ratio Index (PRI) and Delta / Alpha Ratio (DAR)  -Predicting the outcome of rehabilitation with the help of PRI index |
| Saita [45] | Seven patients with chronic stroke | ✓  (n=4) | ✓  (n=3) | 46-68 years | Elbow | Seven weeks (20 sessions of 60 min) | - Extension and flexion movement of elbow joint at least 200 times during each session using the robot | Fugl-Meyer (FMA), Motor Activity Log (MAL), Disability Assessment Scale (DAS), Modified Ashworth Scale (MAS), Barthel Index (BI), Functional Independence Measure (FIM), Functional near infrared spectroscopy (fNIRS), Action Research Arm Test (ARAT), Mini-Mental State Examination (MMSE), Self-Depression Scale (SDS) | - Increasing the cortical activation in the ipsilesional primary sensorimotor area at four-month follow-up according to Fnirs  -The mean ARAT baseline score =15.9 ± 18.3  -Prior to intervention, mean AOU and QOM scores were 0.7 ± 0.9 and 0.7 ± 0.9, respectively.  -BI and FIM scores were 88.6 ± 18.0 and 112.1 ± 24.1 points, respectively.  -Regarding neuropsychiatric assessments, the mean MMSE score was 26.9 ± 3.5, Apathy score was 10.7 ± 6.2 points (score greater than or equal to 14 implicates apathy on the scale)  -SDS score was 43.3 ± 8.2 points representing normal to mildly depress. | -Effectiveness of a combination of highrobot-assisted rehabilitation (RT) and botulinum toxin A (BTX-A) for the treatment of spastic hemiplegia caused by stroke  -Improving motor function after stroke  - Increasing patients' motivation to continue treatment  -Increasing the activity level of the contralesional hemisphere  - Increased activity in the primary sensorimotor area of the ipsilesional hemisphere  - Improves the spasticity  - Increasing neural flexibility |
| Nam[46] | Fifteen patients with chronic stroke | ✓  (n=12) | ✓  (n=3) | 18- 78 years | Hand and elbow | Seven weeks (20 training sessions with the intensity of 3–5 sessions/week) | -Using the robot to perform rehabilitation exercises such as: pulling the elbow joint at an angle of 130 degrees, holding the wrist by a test operator, pulling the thumb up to an angle of about 30 degrees, palmar abduction, bending the elbow and recording muscle activity | Fugl-Meyer Assessment (FMA), Action Research Arm Test (ARAT), Wolf Motor Function Test, Functional Independence Measure (FIM), Modified Ashworth Scale (MAS) | -Significant improvements in the FMA shoulder/elbow and wrist/hand scores (P < 0.05), the ARAT (P < 0.05), and in the MAS (P < 0.05) after the training and sustained 3 months later  - Significant decrease of the muscle activation level in flexor digitorum (FD) and biceps brachii (P < 0.05)  -Significant reduction of CIs in the muscle pairs of FD and triceps brachii and biceps brachii and triceps brachii (P < 0.05) | -Improving voluntary motor performance and muscle coordination in proximal and distal joints with the help of NMES-EMG robotic hand  - Motor improvement after the training could be maintained till 3 months later. |
| McKenzie [47] | Forty patients with stroke | ✓  (n=29) | ✓  (n=11) | Age >18 years | Wrist and fingers | 1-3 weeks | -Moving the wrist and fingers (flexion and extension) with the help of the robot and measuring the movement within 3 degrees of freedom through the robot's sensors | -Fugl-Meyer Arm Motor Scale (FMA), Action Research Arm Test (ART); Box & Blocks test (B/B), Stroke Impact Scale-2 (SIS), Barthel Index (BI), Magnetic Resonance Imaging (MRI) | -Significant correlation between performance on the robot-based tests, including speed (r=0.82, p<0.0001), wrist targeting (r=0.72, p<0.0001), and finger targeting (r=0.67, p<0.0001) with the FMA scores  - Significant correlation between Wrist targeting (r=0.57-0.82) and finger targeting (r=0.49-0.68) with all 5 secondary motor outcomes and with percent CST injury  - Significant correlation between robotic version of the B/B with the clinical B/B test but was less prone to floor effect  -Comparability of Robot-based assessments with FMA score in relation to percent CST injury and superior in relation to M1 hand injury | - Improving the motor function of the upper limb  - Increasing the speed and range of motion of the upper limbs after the intervention |
| Kim [48] | Thirty patients with stroke in two external focus (EF) and internal focus (IF) groups | ✓  (n=16) | ✓  (n=14) | 58.1(12.6) | Arm and shoulder | Four-week (sessions duration from 30 to 90 minutes based on the movement ability of the participant) | -Training arm and shoulder flexion, extension, adduction, and abduction movements through games and performing them with the help of a designed robot | Fugl-Meyer Assessment, and Wolf Motor Function Test | - joint independence EF condition: F1.6,45.4=17.74; P<.0005; partial η2=.39; joint independence IF condition: F2,56=18.66; P<.0005; partial η2=.40; Fugl-Meyer Assessment: F2,56=27.83; P<.0005; partial η2=.50; Wolf Motor Function Test: F2,56=14.05; P<.0005; partial η2=.35 | - Significant improvement in both groups  - No difference in maintenance of motor skills between EF and IF participants four weeks after arm training |
| Bishop [49] | Twelve children with hemiparesis | ✓  (n=5) | ✓  (n=7) | 6-17 years | Hand/finger | Six weeks (3 days per week in 1-hour treatment session blocks) | -Performing range of motion and hand/finger flexion and extension activities by robot | -Assisting Hand Assessment (AHA) test, Jebsen-Taylor Test of Hand Function (JTTHF), Fugl-Meyer scale, Upper Extremity Skills Test (QUEST), Total Active Mobility (TAM), Pediatric Evaluation of Disability Inventory (PEDI) | -Significant improvements after training on the Assisting Hand Assessment (mean difference, 2.0 Assisting Hand Assessment units; P = 0.011) and on the upper-extremity component of the Fugl-Meyer scale (raw score mean difference, 4.334; P = 0.001)  -Don’t significant improvements between pretest and posttest on the Jebsen-Taylor Test of Hand Function, the Quality of Upper Extremity Skills Test, or the Pediatric Evaluation of Disability Inventory after intervention | -Well tolerance training with the hand robotic device by the participants  - Significant improvements in bimanual hand use  -Irrelevance of attentional focus to maintain trained motor skills for people with moderate to severe open weakness  - Importance of exercise dose and intensity to maintain trained motor skills for people with moderate to severe arm weakness |
| Housley [50] | Six stroke survivors with moderate to severe UE impairment | ✓  (n=2) | ✓  (n=4) | 18-85 years | Wrist | Five weeks (2 hours, 3 times per week) | -Performing active and passive wrist range of motion rehabilitation exercises with the robot | Fugl-Meyer Motor assessment, Active active range of motion (AROM) and passive (PROM) range of motion, Wolf Motor Function Test (WMFT), Stroke Impact Scale (SIS) | - Improvement of participants on average on FMA / UE scale (p = 0.05) 5.5 points (21.43%) and previous MCID accreditation between 4.25 and 7.25 points  - - Significant improvements in wrist PROM (+30.52%, p = 0.034) and moderate, no significant  - improvements (+56.15%, p = 0.088) in wrist AROM at postintervention assessment  - Observe the modest, nonsignificant (+3.02%, p = 0.801) improvements in mean WMFT performance times | - Significant clinical improvements in in survivors of moderate to severe stroke through the TDS-HM system  -Perform active, long-term and repetitive rehabilitation exercises using TDS  -Reduction of UE motor defects during the treatment period  - Do more successful work by patients after completing TDS-HM training  -Improving the quality of life in all physical dimensions of SIS for survivors of moderate to severe stroke through TDS-HM intervention  -Improving patients' motor function after training with TDS-HM |
| Hsieh [51] | Thirty-one sub-acute stroke patients divided in two group experimental and control | ✓  (n=18) | ✓  (n=13) | 18 - 80 years | Wrist | Four weeks (90 minutes/day, 5 days/week) | -Experimental group: performing bilateral forearm pronation/supination and wrist flexion and passive and active extension movements with the robot by performing exercises such as filling a bottle from a fountain, transferring to the treatment room and drinking water from the bottle, wiping the table with a cloth and folding the towels and putting them in the drawers  -Control group: Involving reach to grasp, object handling, and pinch and grip movements, and included such works as classification blocks or cards, putting pin into holes, stacking cones, stacking checkers, flipping cards | Fugal-Meyer Assessment, grip strength, and the Box and Block Test, Modified Rankin Scale (MRS), Functional Independence Measure (FIM), Stroke Impact Scale (SIS) | -There was a statistically significant improvement within the group in most outcome measures in both primed and unprimed groups, except activation, after 4 weeks of intervention (all P <0.05).  -Improvement in the modified Rankin Scale (p = 0.065) in the primed group in compared with the unprimed group  -Significantly better improvement on the Stroke Impact Scale strength subscale ( p = 0.012) and a trend for greater improvement on the modified Rankin Scale ( p = 0.065) in primed group than the unprimed group | -Significantly improvement the most outcomes over time in primed and unprimed groups  -Improve perceived muscle strength in the rehabilitation group  - Reduction of disability in stroke patients after intervention |
| Gandolfi [52] | Forty-four patients with MS (experimental group (n = 23) and control group (n = 21)) | ✓  (Not mentioned) | ✓  (Not mentioned) | 18-65 years | Finger | Five weeks (50 min/session, 2 sessions/week) | -Experimental group: Includes (1) continuous passive motion (CPM) to passively stimulate the hand during finger flexion and extension (2) assistive therapy in which the hand has function but is actively trained to the patient's level of function. (3) interactive therapy through active learning with developed virtual therapy games in which the patient exerts isometric force in flexion or extension.  -Control group: including exercises for upper limb mobilization (shoulder girdle, elbow, wrist, and finger joints) | - Action Research Arm test: Assesses upper limb functioning using observational methods, Fugl-Meyer Assessment Motor Scale - Arm Section - Motor Activity Log: Semi-structured interview to assess arm function. - Tremor Severity Scale: A clinical rating scale is a - Nine Hole Peg Test: Measures finger dexterity - Motricity Index: Measures of strength in upper limb - Amadeo hand muscle strength: Measures of muscle strenght using the robotic device   Multiple Sclerosis Quality of Life: Multiple Sclerosis Quality of Life-54 (MSQOL-54) | - No significant difference in primary and secondary results between groups  - No significant difference between groups in ARAT scores  - Significant improvement in performance at T1 and T2 in both groups (P < 0.001)  - Significant increase in UL FMA capacity  - significant changes in the motor activity log-amount of use (MAL-AOU) at both T1 and T2  -At T2 significant improvements in muscle strength during finger extension (p = 0.02) and flexion (p < 0.001) in the EG | - Increasing the use of upper limbs and muscle activity by patients with the help of a robot  - Significant improvement in the body's muscle performance status through robot-assisted training  -Enhancement of extensor carpi activation only in the robot-assisted hand training group  - No side effects of the robot on the body and participants  - Improving skills in the life habits domain (accomplishments) |
| Lee [53] | Nineteen subjects (8 healthy persons and 11 patients with shoulder impairments) | ✓  (Not mentioned) | ✓  (Not mentioned) | Not mentioned | Shoulders | Not mentioned. | -Performing flexion/extension and abduction/adduction movements in sagittal plane and coronal plane using robot | The tracker assesment based on a three-DOF motion capture experiment of the two rehabilitation devices, J-Wrex and CPM, and two basic shoulder motions: flexion/extension and abduction/adduction | -Verification the performance of the device with the two commercially available rehabilitation devices, such as J-Wrex and CPM  - Reducing the interaction force and the distance between the device and the arm by using the robot  - Easier and more effective shoulder rehabilitation through the robot | -Greater range of motion and reduced interaction by the improved tracker  - Creating more natural shoulder movement during rehabilitation tasks by combining the tracker with existing commercial rehabilitation devices. -Tracking of the natural GH movement during upper extremity rehabilitation through the robot |
| Germanotta [54] | Forty-eight patients with sub-acute stroke | ✓  (n=33) | ✓  (n=15) | 40-85 years | Upper limb | Six weeks (daily for 45 minutes, for 5 days per week) | -Performing Grasp, Grip, Pinch and Gross movement using the robot | Fugl-Meyer Assessment of Motor Recovery after Stroke (FMA), Action Research Arm test (ARAT), the Barthel Index (BI).  Test-retest reliability: based on data acquired from patients at the two two-sessions by applying the Intraclass Correlation Coefficient (ICC), using a two-way random outcome, absolute agreement, and multiple measurements model | - Lack of correlation of Barthel Index with robotic indicators  - Strong correlation of FM with robotic indicators  -Weak correlation of Worktot with FM  - Correlation between ARAT and robotic indicators  - Moderate to strong correlation of all presented indicators except Worktot with BI | - Reliable, sensitive and robust all robotic indices for upper limb rehabilitation  - -Effective evaluation of motor function of the upper limb of patients with subacute stroke with the help of a robot |
| Kim [55] | Fourteen patients with hemiplegia | ✓  (n=11) | ✓  (n=3) | 20-85 years | Shoulder | Six weeks | - Carrying out shoulder abduction-adduction and elbow flexion-extension movements in the horizontal plane of the robot | Fugl-Meyer Assessment (FMA), Manual Muscle Test, Hand Function Tests (HFTs), Modified Ashworth Scale (MAS), and Mini-Mental State Evaluation (MMSE), Grip Strength Test, Box and Block Test, and 9-Hole Peg Test (NHPT). | - No change in the non-intervention phase (A1 to A2)  - Increasing the results of FMA, HFT and K-MMSE scores, except for the MAS score in the intervention stage (p < 0.05)  - No significant changes in hand function scores, FMA, MAS and K-MMSE (p>0.05)  - Significant improvement in the mean scores of the mean Hand Grip Strength, Box and Block Test, and NHPT scores from 6.14 (SD 6.63) to 7.29 (SD 7.75) (p = 0.017), from 10.50 (SD9.39) to 15.07 (SD 13.79) (p = 0.008), and from 14.50 s (SD 20.92) to 12.71 s (SD 18.45) (p = 0.043) after training, respectively  - Significant improvement in FMA-UE total score from 30.71±12.96 to 33.64 (SD 15.25) (p=0.001)  - Significant improvement in the total score of proximal and distal sub-scores from 23.36 (SD 6.71) to 25.50 (SD 8.00) (p=0.001) and from 7.50 (SD 7.11) to 8.14 (SD 7.82) (p=0.041)  -Improvement of KMMSE score from 27.36 (SD 2.76) to 28.50 (SD 2.10) (p = 0.013) and MAS score change from 2.07 (SD 0.92) to 2.00 (SD 0.88) (p = 0.317) | - Improving upper limb function and cognition in stroke patients through Neuro-X® training system  -No observation of major or minor side effects during the training period  -Efficacy, low cost, small size and easy control of Neuro-X® for upper limb rehabilitation |
| Villafañe [56] | Thirty-two patients (experimental group (n = 16) and control groups (n = 16) group) | ✓  (n=21) | ✓  (n=11) | 50-90 years | Fingers, shoulder, and arm | Three weeks (30-minute session for 3 day per week for both groups) | -Experimental group: Performing the passive movement of flexion-extension of the fingers by robots  -Control group: Perform exercises such as assisted stretching, shoulder and arm exercises, and functional reaching tasks | National Institutes of Health Stroke Scale (NIHSS), Modified Ashworth Scale, Barthel Index (BI), Motricity Index (MI), Disabilities of Arm, Shoulder, and Hand (DASH), visual analog scale (VAS) measurements | - Significant time factor for NIHSS, BI, MI, and QuickDASH (F1.0 = 94.675; P < .001, F1.0 = 169.731; P = .001, F1.0 = 111.383; P = .001, and F1.0 = 50.063; P = .001, respectively) but not for group-by-time interaction  -No significance for time (F1.0 = 11.791; P = .002), or group-by-time (F1.0 = 0.628; P = .4) for spasticity measured over the MAS  - Significant effect of time (F1.0 = 5.775; P = 0.02) by VAS but not for the group-by-time interaction (F1.0 = 1.444; P = .2) for pain intensity  - - Demonstrated a significant effect of time (F1.0 = 5.775; P = 0.02) by VAS but not for the group-by-time interaction (F1.0 = 1.444; P = .2) for pain intensity  - -A significant effect of time interaction for NIHSS, BI, MI, and Quick DASH, after stroke immediately after the interventions (all, P < .001)  - More pain reduction in the experimental group than in the control group at the end of the intervention, a reduction of 11.3 mm compared with 3.7 mm, using the 100-mm VAS scale | -The effectiveness of the robot in the treatment  -Reduction of pain and spasticity in paralysis of the hand after stroke  - Improvement of hand motor function in stroke patients after intervention  -Safety and reliability of robotic rehabilitation treatment |
| Palermo [57] | Ten subacute stroke survivors | ✓  (n=8) | ✓  (n=2) | 21-83 years | Shoulders, elbow, wrist | Twenty sessions, each lasting 50 min, five sessions per week | -Performing passive movements to familiarize and reduce the patient's spasticity, if any, and 40 minutes of task-oriented exercises | Patient movements: BTS SMART-DX 300 (BTS Bioengineering, Brooklyn, NY, USA; Movement time (MT), Peak velocity (PV), Peak velocity (PV), Normalized Jerk (NJ), Trunk Displacement (TD), Hand Path Ratio (HPR), Time to PV (TtPV) | -Significant differences between pre- and post-treatment kinematic indices MT (Z = −2.701, p = 0.007), NJ (Z = −2.701, p = 0.007), TD (Z = −2.701, p = 0.007), and HPR (Z = −2.701, p = 0.007)  -No significant difference between pre- and post-treatment assessment for PV and TtPV  -No significant difference for PV and TtPV between pre- and post-treatment evaluation  -Significant decrease in clinical evaluation scales FIM (Z = −2.803, p = 0.005), BI (Z = −2.809, p = 0.005), FAT (Z = −2.831, p = 0.005), FMA (Z = −2.807, p = 0.005) before and after treatment  -A strong tendentially significant correlation between FAT and HPR  -A moderate, yet not significant, correlation (0.40 < \|rs\| < 0.59), BI and MT, BI and TD, FAT and TtPV, and FMA and HPR | - Improvement of motor parameters and clinical scales of upper limb disability  -Significant reduction of MT and increase of the patient's re-mobility  -Reducing the time needed to do the exercises  -Increasing the patient's independence in performing therapeutic exercises |
| Iwamoto [58] | Twelve acute stroke patients | ✓  (n=8) | ✓  (n=4) | Group A= 62.33(10.23)  Group B= 59.67 (24.56) | Elbow | Two weeks (40 minutes per day and performed at least 200 movements) | -Performing flexion and extension movement of the elbow joint using the robot | - Functional parameters: motricity index (MI), Grip Strength Test, Modified Ashworth scale (MAS), Functional Independence Measure (FIM), Barthel index (BI), Motor Activity Log (MAL)N Quality of Movement (QOM), Mini-mental state examination (MMSE), Amount of Use (AOU) | - No significant differences in motor function severity as defined by recovery grade (1-12) of hemiplegia (upper limb), Br-stage (upper limb, finger, and lower limb), motricity index, grip strength, Modified Ashworth scale, or sensory function between groups  - No significant difference in mini-mental state examination score between groups  - No significant difference in ADL limitations (FIM-total, FIM-motor subscore, FIM-cognitive subscore, and BItotal) between the groups  - significantly higher score changes of recovery grade (1-12) of hemiplegia (upper limb), FIM-upper body dressing, FIM-motor subscore, BI-dressing, and BItotal during A compared to during B in both group A and group B  - Significantly higher score changes of Br-stage (upper limb and finger), MAL-amount of use (put arm through sleeve), MAL-QOM (put arm through sleeve), and MAL-QOM (total score) during A compared to during B in group A only  - High FIM-total in group A during A (P = .067) and significantly higher during A than during B in group B  - No significant differences in other functional or ADL parameters between groups | -Improvement the upper limb motor function and ADLs, in particular, dressing the upper body during combination HAL-SJ and occupational therapy  -Positive effect of combination HAL-SJ and occupational therapy on ADL function and real use of a hemiparetic arm in the daily life of acute stroke patients |
| Kim [59] | Thirty-six hemiplegic shoulder pain patients | ✓  (n=22) | ✓  (n=14) | Intervention Group=  65.9(9.4)  Control Group= 64.7 (8.3) | Shoulder | Thirty minutes per day, 5 times per week for 4 weeks | -Intervention Group: robotic-assisted shoulder rehabilitation therapy for 30 minutes per day and performing conventional physical therapy exercises to improve upper limb mechanics and reduce nerve damage  -Control Group: Performing conventional physical therapy exercises to improve upper limb mechanics and reduce nerve damage | Fugl-Meyer Assessment (FMA), NIH Stroke Scale (NIHSS), Shoulder Disability Questionnaire (K-SDQ), PROM, Barthel Index asmeasure | - Significant time and group interaction effects on the visual analog scale, in the abduction passive range of motion, and on the Shoulder Disability Questionnaire (F2,33=16.384, P=.002; F2,33=10.609, P=.012; F2,33=32.650, P=.008, respectively)  - Significantly higher improvements in the intervention group than in the control group at T1 after post hoc analysis (P<0.05, all)  - No significant difference between the 2 groups in baseline VAS scores  - -A significant effect of time and group interaction for VAS scores (F2,33Z16.384,PZ.002), after repeated-measures analysis of vari-ance  -Decrease the averageVAS score of the intervention group from 6.60.9 to4.10.7 at T1and sustaine this effect was at T2  - Improve VAS scores more significantly between T1 and T2 in the intervention group than in the control group (tZ 5.491,PZ.003 and tZ 5.282, PZ.002, respectively), after post hoc analysis  - Don't significantly different betweenthe 2 groups in baseline values of the secondary outcome measures, including thePROM of all directions, the K-SDQ, and the sonographic gradesof the affected shoulder  -Show a significant time and groupinteraction effect (F2, 33Z10.609; PZ.012) by PROM in abduction  - Improve the abduction PROM significantly more in the intervention group than in thecontrol group at both T1 and T2 (tZ2.752,PZ.009 andtZ4.534,PZ.012, respectively)  - A significant time and groupinteraction effect (F2,33Z32.650;PZ.008) regarding the K-SDQ  - Decrease the average K-SDQ score from 964to686points at T1 to 656 points at T2 in the interventiongroup  - Significant group differences in the K-SDQ score changesat T1 and T2 (tZ 6.556,PZ.011 andtZ 5.596,PZ.004,respectively) | -Improves hemiplegic shoulder pain and self-reported shoulder-related disability  - Performing joint mobili-zation and stretching exercises of the shoulder in patients in asupine position by robot  - Decrease pain due to HSP in stroke patients  - improves the PROM of the affected shoulder and lower self-reported shoulder-related disability  - The usefulness of the robot in augmenting current therapy for HSPpoststroke  - |
| Dehem [60] | Forty-five patients with acute stroke into 2 groups (conventional therapy, n=22, and robotic-assisted therapy (RAT), n=23) | ✓  (n=21) | ✓  (n=24) | Age>= 18 years | Upper limb | Nine weeks (four sessions/week) | -RAT: moving the hand along a reference path (e.g., golf course) while passing checkpoints (e.g., golf balls) in a game by robot  -Conventional therapy: Performing rehabilitation exercises without the use of a robot | Box and Block test, Wolf Motor Function test, Stroke Impact Scale | - Improved gross manual dexterity (Box and Block test +7.7 blocks; P = 0.02), upper-limb ability during functional tasks (Wolf Motor Function test + 12%; P = 0.02) and patient social participation (Stroke Impact Scale +18 %; P = 0.01)  - Improving manual activities and activities of daily living in both groups | - Improve hand skills, upper limb ability during functional tasks, and patient social participation  - Effectiveness of robotic-assisted therapy (RAT) compared to conventional therapy  - Improving gross manual dexterity, UL ability during functional tasks and patient's social participation in the early rehabilitation phase with the help of RAT |
| Hung [61] | Forty-four patients with stroke (Robot-assisted therapy  (RT)=15; unilateral hybrid therapy (UHT)=14; bilateral hybrid therapy (BHT)=15) | Not mentioned | Not mentioned | UHT =53.17)12.28(  BHT= 58.45±)13.11(  RT=52.68)8.75 | Forearm | Six weeks (90 min/d 3 d/wk) | -BHT: Performing rehabilitation exercises of forearm pronation-supination and wrist flexion-extension using a robot  -UHT: Performing rehabilitation exercises of forearm pronation-supination and wrist flexion-extension using a robot and frequent use of the arm (such as eating and dressing)  -RT: Performing forearm supination/pronation rehabilitation exercises actively and passively using the robot | Fugl-Meyer Assessment (FMA), Stroke Impact Scale (SIS), Wolf Motor Function Test (WMFT), Nottingham Extended Activities of Daily Living (NEADL) | -Favor BHT over UHT on the FMA total score and distal score at the posttest (P = .03 and .04) and follow-up (P = .01 and .047) assessment and BHT over RT on the follow-up FMA distal scores (P = .03)  -Significant improvement in the WMFT and SIS scores of the 3 groups without between-group differences in posttest assessment  - Significant greater improvement in the mobility domain of NEADL compared to the BHT group (P <.01) | .- Improves the upper limb motor function, especially distal motor function in patients after robot-assisted therapy (RT)  -Immediate effects and differential persistence in stroke rehabilitation by hybrid therapy and RT  -unilateral hybrid therapy (UHT) as an opportunity to increase physical ability related to UE and QOL  -RT and UHT are promising solutions to promote IADL independence, especially in the area of pro-RT mobility |
| Conroy [62] | Forty-five participants (robot therapy (RT): n = 22) and transition-to-task training (TTT) n = 23)) | ✓  (n=29) | ✓  (n=16) | 56.1(11.4) | Shoulder and elbow | Twelve-week (1 hour, 3 times a week) | - RT:Performing 1132 repetitions in block 1 (wrist robot training), 1539 repetitions in block 2 (shoulder elbow training) and 1443 repetitions in block 3 (intermittent robots) in RT group  -TTT: Performing 724, 1118 and 958 repetitions of each session in the same training blocks of the RT group | Wolf Motor Function Test (WMFT) and Stroke Impact Scale (SIS), UE FMA proximal | - No significant 12-week difference in FMA change between groups, and mean FMA gains were 2.87 ± 0.70 and 4.81 ± 0.68 for RT and TTT  - No significant difference between the 2 robot therapies and did not support the hypothesis based on FMA  Improved further 12-week secondary outcome of TTT in WMFT log (-0.52 ± 0.06 vs -0.18 ± 0.06; P = .01) and SIS hand (20.52 ± 2.94 vs 8.27 ± 3.03; P = .03) | - Improve the motor performance indicators (WMFT) and self-reported stroke-affected hand use (SIS) without loss of benefit from less time on the robot |
| Bonanno [63] | A woman with relapsing-remitting multiple sclerosis (RRMS) | - | ✓  (n=1) | 47-year-old | Fingers | One month (40 1 hour-training sessions (i.e., 5 times a week for 8 consecutive weeks)) | -Performing the opposite movements of the finger (thumb to index finger-middle ring-small ring) respectively with your right and left hand for 180 seconds in a row (60 active movements - 60 rest positions) | Functional MRI (fMRI), Nine Hole Peg Test | - Significative improvement GAF and fMRI evaluation, parameters at the end of the rehabilitation program  - Demonstrate a significant increase in functional activation in the motor-sensory network in active and motor work by fMRI | - Proof of possible restorative effect of robotics on brain networks  - AF as a valuable tool in assessing functional recovery after upper limb rehabilitation, especially of associated to fMRI examination  - Improve clinical signs after 1 month of robot-based training |
| Leem [64] | Forty-eight hemiplegic patients | ✓  (n=30) | ✓  (n=18) | 18-85 years | Wrist and fingers | over 4 weeks (5 days per week for 30 minutes per day) | -Robot-assisted therapy exercises are designed to stimulate targeted movements of the wrist and fingers, including forearm supination/pronation, wrist flexion/extension, wrist radial/ulnar flexion, and finger flexion/extension. | Modified Ashworth Scale (MAS), Mini-Mental State Examination (MMSE), functional independence measure (FIM) , Manual Function Test (MFT) | - Significant changes in mean scores between the baseline and post-treatment in the MFT and FIM  - Correlation age and initial MMSE (r = 0.54) with the final FIM, and the MAS-UE (r = −0.54)  -Correlation MFT-A (r = 0.80) with the final MFT  - OTR (P = 0.034) as the significant predictors of the final MFT  - MMSE (P < 0.001) as a significant predictor of the final FIM, and MAS-UE (P = 0.022), initial MFT-A (P < 0.001) | - less spasticity, better initial cognitive function, and better initial motor function after robot-assisted therapy  - clinically significant motor function after upper limb Robot-assisted therapy (RT) |
| Kim [65] | Thirty patients in tow groups (rtual reality rehabilitation group (SMVR) n=15; and control group (CON) n=15) | ✓  (n=14) | ✓  (n=16) | SMVR= 59.4(1.8)  CON= 54.73(2.98) | upper limb active joint angle | Eight weeks (60min a day, three times a week) | - SMVR: performing 60 minutes three times a week for 8 weeks in upper extremity sensory stimulation and robot virtual reality rehabilitation  - CON: Performing conservative treatment and peripheral joint movement for 60 minutes | Jebsen-Taylor hand function test (JTT), Stroop test (ST) and Trail making test (TMT) | -A significant difference (P<0.05) between before and after training in both groups, significant improvement in both groups (showed by SMVR group)  -Existence of groups Significant differences in the active joint angle and function of the upper limb as well as the ability to concentrate before and after training within the groups (p <0.05)  -More significant improvement in the SMVR group than in the CONgroup (p<0.05), except for FPAROM and JTT | - Confirmation of the positive effect of robot virtual reality training on limb motion stimulation  -Improvement the range of motion, active joint angle, function, and concentration of upper extremity active joints  - Positive effect on the ability to concentrate in chronic stroke patients |
| Tartamella [66] | A patient with BRN | ✓  (n=1) | - | 57-year-old | Shoulde, elbow, wrist and finger | Two-month (6 days a week, a daily 180-minutes) | -Performing rehabilitation exercises related to the shoulder, elbow, wrist and finger muscles and joints, pronation, supination, wrist flexion and extension, and grasping and releasing using the robot. | Functional Independence Measure (FIM), Modified Ashworth Scale (MAS), Mini-Mental State Examination (MMSE), Muscle Research Council (MRC), Magnetic Resonance Imaging (MRI), Performance Oriented Mobility Assessment (POMA), Reliable Change Index (RCI) | -Good patient tolerance of the entire robot-assisted training program  - Significant improvement in upper limb functional disability in four dimensions of spasticity, degree of cognitive impairment and balance | - Usefulness of neurorobotic intensive rehabilitation in BRN to reduce functional disability  -Significant improvement in functional independence and clinical status of patients  - No side effects caused by the robot to the patient (including falling, muscle/tendon/joint stretching, skin irritation) |
| Solaro [67] | Forty-one clinically definite MS subjects with upper limb impairment (rbot-based haptic training (‘Haptic’) or purely sensorimotor training (‘Sensorimotor’) groups) | ✓  (Not mentioned) | ✓  (Not mentioned) | Age >=18 years | Hand | Eight training sessions (40 min/session, 2 sessions/week) for both groups | - Haptic group: Hand movement in six directions (0°, 60°, 120°, 180°, 240°, 300°)  - Sensorimotor group: Performing sensorimotor rehabilitation exercises without using a robot | Active and Passive Range Of Motion (ROM), Patient | -Decreasing the average score of 9HPT from 9±74 s to 8±61 s for the Haptic and from 6±49 s to 6±44 s  - 9HPT improvement (decrease after treatment) in 15/19 (79%) subjects in the Haptic group and in 10/17 (59%) subjects in the Sensorimotor group  - In terms of response to treatment, 6/19 (32%) responding subjects in the Haptic group (3 PYR, 2 CBL, 1 MIX) and 3/17 (18%) in the Sensorimotor group  ی- absolute change was only significant in the Haptic group (p=.011)  - the Sensorimotor group exhibited a better initial performance than the Haptic group  - The ARAT score increased from 47 ± 1 to 50 ± 1 in the Haptic group, and from 51 ± 1 to 53 ± 1 in the Sensorimotor group  -4/6 subjects in the Haptic group and 2/3 in the Sensorimotor group showed an improvement in the ARAT score  - No fatigue of the participants at the end of each session | -Improving the function of the upper limbs of patients  - No side effects for the patient  - Safety of the robot for patients |
| Picelli [68] | Twenty adult outpatients with distal radius fracture due to wrist injury (Robotic Arm Training (RAT) group and Conventional Arm Training (CAT) group) | ✓  (n=7) | ✓  (n=13) | Age >=18 years | Wrist and forearm | Ten, 1-hour (40 minutes of arm training + 20 minutes of conventional occupational therapy) | -RAT: receiving arm training by means of a robotic device and performing exercises such as pronation/supination of the forearm and extension/flexion of the wrist according to three methods: passive-passive (both arms are moved by the robot), active-passive (one arm guides the other arm) and active-active (Both arms actively move in front of each other)  -CAT: performing arm training following a conventional rehabilitation program) trainings such as active function and strength of forearm pronator/supinator and wrist extensor/flexor ( | Active and Passive Range Of Motion (ROM), Patient | - No significant differences in terms of primary (wrist range of motion) and secondary (pressure applied to the hand, wrist, and hand scores assessed by the patient) at all-time points  - As to the PROM, no significant difference between the two groups were found at T1–T0 as to forearm pronation (P = 0.927; Z = -0.091; effect size = -0.05) and supination (P = 0.750; Z = -0.319; effect size = 0.18) as well as to wrist extension (P = 0.172; Z = -1.366; effect size = 0.42) and flexion (P = 0.636; Z = -0.473; effect size = 0.15)  - No significant difference between the two groups were found at T2–T0 as to forearm pronation (P = 0.927; Z = -0.091; effect size = -0.05) and supination (P = 0.459; Z = -0.740; effect size = 0.15) as well as to wrist extension (P = 0.269; Z = -1.105; effect size = 0.21) and flexion (P = 0.064; Z = -1.850; effect size = 0.48)  - With regard to the AROM, no significant difference between the two groups were found at T1–T0 as to forearm pronation (P = 0.701; Z = -0.383; effect size = 0.07) and supination (P = 0.426; Z = -0.795; effect size = 0.27) as well as to wrist extension (P = 0.460; Z = -0.739; effect size = 0.26) and flexion (P = 0.288; Z = -1.063; effect size = 0.25).  -No significant difference between the two groups were found at T2–T0 as to forearm pronation (P = 0.701; Z = -0.383; effect size = 0.07) and supination (P = 0.337; Z = -0.961; effect size = 0.20) as well as to wrist extension (P = 0.161; Z = -1.402; effect size = 0.35) and flexion (P = 0.268; Z = -1.107; effect size = 0.29).  - As to the PRWHE score, no significant difference between the two groups were found at T1–T0 (P = 0.207; Z = -1.261; effect size = -0.36) and T2–T0 (P = 0.094; Z = -1.686; ; effect size = -0.48) | - Robot arm training as a practical tool for the treatment of upper limb disorder in adult patients with distal radius fractures  -Use a robot-assisted arm to increase therapist focus and simultaneously monitor more than one patient in rehabilitation sessions |
| Kuo [69] | Seven children with CP | ✓  (n=6) | ✓  (n=1) | 6-18 years | Fingers | Six weeks (12 -60-min sessions, 2 times a week) | - Warm-up exercises (included weight-bearing and rhythm activities) and 40 min of finger training by robot | Fugl-Meyer Assessment-Upper Extremity (FMA-UE), electromyography for muscle activity, box and block test (BBT), Grip Strength Dynamometer Test, and ABILHAND-Kids | -Significant improvement in the mean amplitude of the brachioradialis muscle (P = 0.015) and the ratio of the agonist-electrical antagonist muscle (P = 0.041) in the 1-inch cube-grasping task  - Significance of time effect in FMA-proximal, FMA-distal and FMA-total components (mean improvement of 5.72, 3.14 and 8.86, respectively, all P = 0.002, respectively)  -Significance time effect on the mean BR muscle amplitude (55.43 ± 30.34 to 39.29 ± 17.79; P = 0.015) and EAA ratio (0.56 ± 0.10 to 0.50 ± 0.08; P = 0.041) during task  - Observed a change in CR during the maximal voluntary hand opening and maximal grasping (0.73 ± 0.09 to 0.67. 0.12; P = 0.084 and 0.34. 0.11 to 0.41. 0.13, respectively; P = 0.084).  - Increase the ED amplitude area by 57.14% (4/7, range 23.95-104.72%) in the hand opening movement  - Increase the ED amplitude area by 57.14% (4/7, range 23.95-104.72%) in the hand opening movement  - Increase the BR amplitude area by 71.43% (5/7, ranged 9.14-424%)in the grasping movement  -Minor improvements in BBT scores and insignificant time effect (mean score improvement 1.71; P = 0.170)  - Decrease the mean BR amplitude by 71.43% (5/7), 71.43% (5/7), and 57.14% (4/7), respectively (range, 2.57-72.64%, 10.19%-76.22%, and 15.28- 90.42%, respectively) in tasks 2, 3, and 4 | -Significant improvement in upper limb function with robot-assisted training (RT  -Lack of improvement in the patient's ability to perform activities and participation in treatment  -Improving RT using the Gloreha device on body structure and function, including excellent motor function, recruitment and coordination of BR muscles in children with CP  . |
| Aprile [70] | Two hundred and twenty-four  patients with stroke | ✓  (n=127) | ✓  (n=97) | 40-85 years | Shoulder | Three months (30 rehabilitation sessions, 45 minutes daily) | -Performing rehabilitation exercises prescribed by the therapist using a set of robotic and sensor-based devices (Motore, Humanware; and Amadeo, Diego and Pablo, from Tyromotion) | Fugl–Meyer Assessment for Upper Extremity, Motricity Index, Modified Barthel Index, Short Form Health Survey (SF-36), Physical health Composite Score (PCS), Mental health Composite Score (MCS), Numeric Rating Pain Scale (NRPS), and Douleur Neuropathique 4 (DN4) | - Pain negative correlation at baseline was with the improvement of upper limb motor function  -Shoulder pain after moderate / severe stroke in 28.9% of patients  -Significant reduction in moderate / severe pain and neuropathy component after both treatments and maintenance of this reduction in T2  -Increased pain intensity in women and in patients with neglect syndrome  -Not significant interaction factor time group, neither considering the whole sample (N ¼ 121, p ¼ 0.961), nor in the subgroup analysis (NRS <5: N ¼ 88, p ¼ 0.987; NRS 5: N ¼ 33, p ¼ DN4þ: N ¼ 27, p ¼ 0.977)  - No differences detection for NRS average scores (p ¼ 0.816) with concerning the main effect of time  - Clinically relevant reduction of pain over time (p < 0.001) | - Improving motor function, strength, and the physical aspects of the quality of life  - Reducing shoulder pain in patients |
| Aprile [71] | Fifty-one patients with stroke | ✓  (n=29) | ✓  (n=22) | 68.4 (12.4) | Upper limb | 30 rehabilitation sessions, each session lasting 45 minutes, 5 days a week | -Performing therapeutic exercises such as driving, washing dishes and collecting coins in games through the robot | Symbol Digit Modalities Test, Digit Span Task, Rey Osterrieth complex figure test (ROCF), Stroop Color and Word Test (SCWT), Oxford Cognitive Screen (OCS), Symbol Digit Modalities Test (SDMT), Tower of London test, Stroop Color and Word Test (SCWT), Fugl–Meyer Assessment, Motricity Index and modified Barthel Index | - Significantly reduction the percentage of patients obtaining a pathological score in the OCS subscore in the episodic memory (p = 0.008), calculation (p = 0.021), and visual attention (heart cancelation task, p = 0.001) fields, after treatment  - A statistically significant improvement was in all the investigated domain: attention and processing speed (Symbol Digit Modalities Test), memory (Digit Span score), visuospatial abilities and visual memory (Rey–Osterrieth complex figure), and executive functions (Stroop errors and time, Tower of London error and time)  - Don’t significantly change “points” of the Tower of London Test did  - A significant improvement in upper limb impairment, measured using the Fugl–Meyer Assessment (mean change, 11.9 ± 10.1; p < 0.001); upper limb muscle strength, as measured by the Motricity Index (mean change, 16.2 ± 12.9; p < 0.001); and ability in activities daily living, as shown by the modified Barthel Index (mean change, 22.6 ± 15.5; p < 0.001), after the treatment | - Improving the patient's condition in all cognitive areas examined after treatment  - Improving the patient's attention and processing speed, memory, visuospatial abilities and visual memory, and executive functions after treatment  - Significant improvement in upper limb muscle strength and ability in activities daily living |
| Bouteraa [72] | A patient with stroke | Not mentioned. | Not mentioned. | Not mentioned. | Arm, wrist, forearm | Two weeks (Exercise 5 times with 50 repetitions per day) | - Performing therapeutic exercises such as flexion/extension, pronation/supination of the arm, , forearm and wrist using a robot | Range of motion (RoM), EMG assessment | -Range of motion (Elbow (flection=140, Extension=50, Supination=0, Pronation=80) and Shoulder (Flection= 180, Extension=50) )  -Wrist range of motions (Palmar flexion=70, Dorsal flexion= 90, Radial abduction= 20, Ulnar abduction= 40)  -Frequencies of stimulation modes (Pain relief= 80, Massage= 80 (3 sec) and 2 (2 sec), Relax= 80 (3 sec) and 2 (2 sec)) | -Improve the quality of the rehabilitation process  -The integration of neuromuscular electrical stimulation into the physical rehabilitation process for effective rehabilitation sessions for neuromuscular recovery of the upper limb  - Pain relief, massage and relaxation  - Set the exercise movement parameters, define the stimulation mode and record the patient training in real time by physiotherapist |
| Kim [73] | Thirty participants with chronic stroke | ✓  (n=14) | ✓  (n=16) | 58.1(5.1) | Arm | Over 8 weeks (12 sessions of standardized robotic proximal arm training on the InMotion ARMa) | -Twelve standardized proximal robotic arm training sessions at InMotion ARM over four weeks | Motion ARMa, Fugl-Meyer Assessment - Upper Extremity (FMA-UE), Wolf Motor Function Test – Functional Ability Scale (WMFT-FAS) | -Confirmation of moderate to severe upper extremity disorder based on baseline score means in FMA-UE 18.7 and 1.8 in WMFT-FAS  -Adherence to the instructions at 0.38 points per week (The average adherence score was 5.7 + 3.3 and the rate of adherence change was .38+ .24)  -Transfer the FMA-UE scores to normal distribution by square root and WMFTFAS scores  - Statistically significant effects (all p’s < .001) on both outcomes at Week 4 and Week 8  -Don't significance the effects of average adherence and rate of adherence over time (all p’s > .2) | - Improvement of motor function after 12 wks of robotic training |
| Bui [74] | Twenty-one subjects with cognitive and upper limb motor impairments | ✓  (n=13) | ✓  (n=8) | Age>= 18 years | Upper limb | Trajectory Tracking Motor Task= 15 seconds and 15 times after one training trial | Performing rehabilitation exercises such as moving blocks and placing them in a box and inserting all the grooved clips into matching holes on a board using the robot. | - Box and Blocks Test (BBT), Grooved Pegboard (GP), and grip strength | - The average sequence length on the robot-based spatial span task varies from person to person  - Observe the strong correlations between robot-based measures and cognitive and motor clinical assessments related to the HIV population (Color Trails 1 (rho = 0.83), Color Trails 2 (rho = 0.71), Digit Symbol - Coding (rho = 0.81), Montreal Cognitive Assessment - Executive Function subscore (rho = 0.70), and Box and Block Test (rho = 0.74)) | -Improving HIV-related motor and cognitive disorders |
| Flynn [75] | Twenty-five stroke survivors | ✓  (Not mentioned) | ✓  (Not mentioned) | Not mentioned. | upper limb | Twelve months | Not mentioned. | Comparison of double-audit records for the number of primary survivors of subacute stroke using: RT-UL, the number of RT-UL sessions, duration of RT-UL sessions, and disciplines prescribing RT-UL | -Reduce the total number of RT-UL sessions between audits (148 vs. 36 sessions)  -Overall reduction in admission rates for stroke survivors  - No significant difference between audits in the average number of RT-UL sessions per patient (p = 0.203) nor the length of sessions (p = 0.762)  - Use of robotic devices by active treated patients for more than three quarters of the time  - Physiotherapists as the primary prescribers of RT-UL when compared to occupational therapists | -Continuation in performing rehabilitation exercises with the help of a robot |
| Terranova [76] | Fifty-one patients with mild to moderate upper limb impairment | ✓  (n=26) | ✓  (n=25) | Age>= 18 years | Shoulder, elbow, wrist | Twelve weeks (three weekly sessions of 60 min) | -Performing shoulder and elbow, wrist rehabilitation exercises with the help of a robot | Wolf Motor Function Test (WMFT), Fugl-Meyer Assessment Upper Limb (FMA-UL) | -No statistical difference between the two groups due to p values of mean change in function measured by WMFT and FMA 0.293 and 0.187  - A significant improvement for WMFT and FMA-UL  -The mean change, measured by FMA-UL for CIMT and RT: 4.5 points for CIMT and 2.7 points for RT (p = 0.187).  -The mean change, measured by FMA-UL for CIMT and RT regarding WMFT: −24.36 and −11.09 s for CIMT and RT, respectively (p = 0.293). | - Significant improvement in upper limb function, motor recovery, functionality, and activities of daily living after Robotic Therapy (RT) intervention |
| Shi [77] | Sixteen chronic stroke subjects | ✓  (n=11) | ✓  (n=5) | 54.9 (17.3) | Fingers, wrist and elbow | Twenty sessions of 1-hour (with the intensity of 3 sessions per week) | -Performing movement exercises of wrist bending and extension, elbow supination and pronation, and finger flexion and extension with the help of the robot | Action Research Arm Test (ARAT), Fugl-Meyer Assessment for Upper Extremity (FMA-UE), Box-and-Block test (BBT), Modified Ashworth Scale (MAS), Maximum Grip Strength (GRIP) | - Significant improvement of upper limb function in ARAT (increased mean=2.44, P = 0.032), BBT (increased mean=1.81, P = 0.024), FMA-UE (increased mean=3.31, P = 0.003), and maximum voluntary grip strength (increased mean=2.14 kg, P < 0.001) for all participants  - No significant change in terms of spasticity with the MAS (decreased mean=0.11, P = 0.423)  - Significant improvement of upper limb function in subjects with mild or no finger flexor spasticity (MAS<2, n = 9) after 20 sessions of training  - No significant change in clinical scores for subjects with moderate and severe finger flexor spasticity (MAS=2,3, n = 7) at pre-training  - Significant increase of maximum voluntary grip strength for subjects with moderate and severe finger flexor spasticity (MAS=2,3, n = 7) | - Significantly improvement for motor function in terms of the performance in ARAT, FMAUE, FMA-WH, and BBT |
| Chen [78] | Twenty stroke patients | ✓  (n=15) | ✓  (n=5) | 18–80 years | Arm | Four weeks (45-min training daily, 5 days/week) | -Performing movement exercises in the 3D workspace with 7 degrees of freedom including shoulder (flexion/extension, abduction/adduction and internal/external), elbow (flexion/extension, forearm supination/pronation) and wrist (flexion/extension and ulnar deviation/ radial deviation) using the robot | Behavioral Inattention Test (BIT), Catherine Bergego Scale (CBS), Fugl-Meyer Assessment for Upper Extremity (FMA-UE), Modified Barthel Index (MBI), and World Health Organization Disability Assessment Schedule (WHODAS) | - Significantly greater improvements in FMA-UE (difference, 5.10; 95% CI, 1.52–8.68, P = 0.01), BIT-C (difference, 7.70; 95% CI, 0.55–14.85, P = 0.04), and WHODAS 2.0 (difference, −7.30; 95% CI, −12.50 to −2.10, P = 0.01) by RAT therapy  - Significant improvements in neglect symptoms assessed by BIT-C in both groups  - Significantly improvements in BIT-C (difference, 7.70; 95% CI, 0.55–14.85, P = 0.04) than the CT group in participants assigned to RAT group  - No significance between the groups (difference, −1.30; 95% CI, −2.89 to 0.76, P = 0.10) regarded to the change scores of CBS  - Better mean Fugl-Meyer scores for measuring upper limb movement disorder for the RAT group than the CT group (difference, 5.10; 95% confidence interval (CI), 1.52-8.68, P = 0.01) after the 4-week intervention  - Improvement in ADL RAT and conventional training with regard to MBI in  - Significant improvement in social participation on change scores of the WHODAS 2.0 (difference, −7.30; 95% CI, −12.50 to −2.10, P = 0.01) in participants receiving RAT therapy than CT group | -Significant improvements in all outcome measures(in neglect symptoms, motor function recovery, and social participation) in both groups |
| Qu [79] | Six (One healthy adult and five stroke patients) | ✓  (n=5) | ✓  (n=1) | 56–75 years | Upper limb | Four-week (more than three times per week for a total of 15 therapy days) | -Using a robot to perform shoulder and elbow rehabilitation exercises | FMA-UE, Modified Barthel index (MBI) , Mini-Mental State Examination (MMSE) | - Significant improvements in MBI and Fugl-Meyer scores of the five patients after the intervention  - Increases the scores of patients 1, 2, 3, 4, and 5 to 5, 7, 7, 7, and 3 after the fifth training session, respectively  - Increases the scores of the patients by 12, 9, 13, 10, and 14 for subjects 1, 2, 3, 4, and 5, after the fifteenth training session, respectively  -An upward trend in Fugl-Meyer SEC scores for the five subjects  - Increases the MBI scores for the five patients  - Increases the patients' scores for the second evaluation from 0 to 13, being 0, 13, 9, 3, and 6 for subjects 1, 2, 3, 4, and 5, respectively  - Increases the scores ranged from 10 to 30, being 13, 30, 25, 10, and 21 for subjects 1, 2, 3, 4, and 5, for the last assessment, respectively | -Safe and effective designed robot  -Facilitate the recovery of stroke patients' UL functiony designed robo |
| Abd [80] | Forty individuals with chronic stroke | ✓  (n=27) | ✓  (n=13) | 50 to 60 years | Upper limb | Twelve successive weeks (with three sessions per week) | -Using the robot to perform rehabilitation exercises such as shoulder flexion, shoulder abduction, shoulder external rotation, wrist extension, handgrip | Action Research Arm Test (ARAT), Wolf Motor Function Test (WMFT), Modified Ashworth Scale (MAS), Active Range of Motion (AROM), Handgrip Strength (HGS) | - Reduce the total number of RT-UL sessions between audits (148 vs. 36 sessions)  -Reduction in admission rates for stroke survivors  - No significant difference between audits in the average number of RT-UL sessions per patient (p = 0.203) nor the length of sessions (p = 0.762)  -- Physiotherapists as the primary prescribers of RT-UL when compared to occupational therapists | - RT-UL as a sustainable and intensive intervention for stroke survivors within an inpatient rehabilitative setting  - Modulating spasticity and improving the motor functions of the affected upper limbs by training with robot-mediated virtual reality gaming |
